# The Gcn4 transcription factor reduces protein synthesis capacity and extends yeast lifespan

**DOI:** 10.1038/s41467-017-00539-y

**Published:** 2017-09-06

**Authors:** Nitish Mittal, Joao C. Guimaraes, Thomas Gross, Alexander Schmidt, Arnau Vina-Vilaseca, Danny D. Nedialkova, Florian Aeschimann, Sebastian A. Leidel, Anne Spang, Mihaela Zavolan

**Affiliations:** 1 Computational and Systems Biology, Biozentrum, University of Basel, Klingelbergstrasse 50-70, 4056 Basel, Switzerland; 2 Growth and Development, Biozentrum, University of Basel, Klingelbergstrasse 50-70, 4056 Basel, Switzerland; 3 Proteomics Core Facility, Biozentrum, University of Basel, Klingelbergstrasse 50-70, 4056 Basel, Switzerland; 4 Max Planck Research Group for RNA Biology, Max Planck Institute for Molecular Biomedicine, Von-Esmarch-Strasse 54, 48149 Münster, Germany; 5 Friedrich Miescher Institute for Biomedical Research, Maulbeerstrasse 66, 4002 Basel, Switzerland; 6 Cells-in-Motion Cluster of Excellence, University of Muenster, 48149 Muenster, Germany; 7 Faculty of Medicine, University of Muenster, Albert-Schweitzer-Campus 1, 48149 Muenster, Germany

## Abstract

In *Saccharomyces cerevisiae*, deletion of large ribosomal subunit protein-encoding genes increases the replicative lifespan in a Gcn4-dependent manner. However, how Gcn4, a key transcriptional activator of amino acid biosynthesis genes, increases lifespan, is unknown. Here we show that Gcn4 acts as a repressor of protein synthesis. By analyzing the messenger RNA and protein abundance, ribosome occupancy and protein synthesis rate in various yeast strains, we demonstrate that Gcn4 is sufficient to reduce protein synthesis and increase yeast lifespan. Chromatin immunoprecipitation reveals Gcn4 binding not only at genes that are activated, but also at genes, some encoding ribosomal proteins, that are repressed upon Gcn4 overexpression. The promoters of repressed genes contain Rap1 binding motifs. Our data suggest that Gcn4 is a central regulator of protein synthesis under multiple perturbations, including ribosomal protein gene deletions, calorie restriction, and rapamycin treatment, and provide an explanation for its role in longevity and stress response.

## Abstract (web summary)

The transcription factor Gcn4 is known to regulate yeast amino acid synthesis. Here, the authors show that Gcn4 also acts as a repressor of protein biosynthesis in a range of conditions that enhance yeast lifespan, such as ribosomal protein knockout, calorie restriction or mTOR inhibition.

## Introduction

The discovery that individual genes can significantly and reproducibly modulate the lifespan of eukaryotic organisms^1^ opened the process of aging to investigation by geneticists and molecular biologists. The ease of its genetic manipulation has made the yeast *Saccharomyces cerevisiae* an important experimental model for aging studies. The number of divisions that a mother yeast cell undergoes before it enters senescence is used as a measure of lifespan, also called replicative lifespan^2^. Genetic studies have linked nutrient sensing pathways to aging (extensively reviewed in refs. ^3, 4^) and consistently, caloric restriction improved the functionality and increased the lifespan of many model organisms (reviewed in ref. ^5^). Although the molecular mechanisms underlying changes in lifespan are still debated^4^, modulation of protein synthesis by the target of rapamycin (TOR) serine/threonine kinase seems to play an important role^6, 7^. Reducing TOR activity increased lifespan in yeast, worms, flies, and mammals^8^. Furthermore, deletion of translation-related genes such as *SCH9*, *TIF1*, and *TIF2*, increased the yeast replicative lifespan^9–11^, and inhibition of translation in the worm *Caenorhabditis elegans* promoted longevity^12–14^. These observations indicate that reducing cellular translation is a conserved mechanism of lifespan extension^15^.

Translation of messenger RNA (mRNAs) into proteins is carried out by the ribosome, a molecular machine that in *S. cerevisiae* is composed of four ribosomal RNAs and 78 ribosomal proteins (RPs). The yeast genome contains 137 RP-encoding genes, 59 of which are paralogs^16^. Screening studies have found that deletion of *RPL31A*, *RPL6B* and other RPs increased replicative lifespan^9, 10, 17^, and a systematic survey of 107 RP gene deletion strains comprehensively showed that the specific reduction of the 60S ribosome subunit significantly extends replicative lifespan^18^. The fact that RPs are downstream targets of TOR signaling reinforces the link between nutrient sensing pathways, protein synthesis, and aging^18–20^.

Lifespan extension in large ribosomal subunit protein (RPL) deletion strains depends on the upregulation of Gcn4^18^. This protein is the key transcriptional activator of amino acid biosynthesis genes in yeast, being translationally upregulated in various stress conditions^21–25^, as well as upon deletion of RPL genes^18, 26, 27^. Other modulators of aging such as the tRNA transporter Los1 and mitochondrial AAA protease gene Afg3 are also thought to exert their lifespan-increasing effects through Gcn4^11, 28^. The *GCN4* mRNA provides one of the best well characterized models of translational control^29^. When sufficient nutrients are available, most ribosomes are sequestered at four upstream open reading frames (uORFs) present in the 5′ UTR, resulting in low Gcn4 abundance. Upon amino acid starvation, the Gcn2 kinase phosphorylates the translation initiation factor eIF2α, leading to reduced levels of GTP-bound eIF2 and depletion of the ternary translation initiation complex. This allows the scanning of 40S ribosomes past the uORF4, resulting in increased initiation at the main ORF and higher production of Gcn4. However, in spite of Gcn4’s central role in yeast longevity, it is difficult to link mechanistically its activity of transcriptional activator of amino acid biosynthesis genes to lifespan extension.

Characterizing the gene expression of RP deletion (RPKO) strains with mRNA sequencing (m RNA-seq), ribosome profiling and proteomics we found that reduced protein synthesis, impaired ribosome assembly and general uORF skipping leading to Gcn4 expression are hallmarks of RPKO strains with increased replicative lifespan. Consistently, we show that Gcn4 is necessary for the translation repression observed not only in long-lived RPKO strains, but also under glucose starvation and rapamycin treatment conditions, and that overexpression of Gcn4 is sufficient to promote longevity and reduce protein biosynthesis. Our results thus suggest that the reduction in protein synthesis capacity contributes to the Gcn4-mediated lifespan extension.

## Results

### Long and short-lived RPKO strains differ in gene expression

**Fig. 1:**
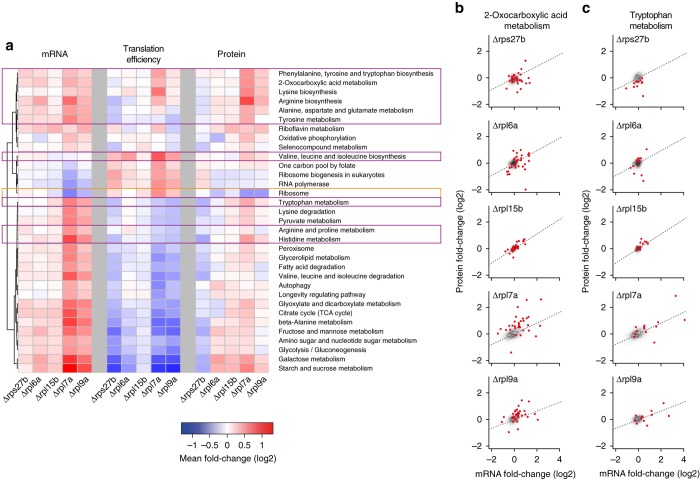
RPKO strains exhibit changes at multiple levels of gene expression. **a** Heatmap depicts mean fold-changes of components of the indicated KEGG pathways at mRNA (measured by mRNA-seq), translation efficiency (calculated by dividing the normalized read count in Ribo-seq by the normalized read count in mRNA-seq), and protein (measured by proteomics) levels. The *orange box* highlights ribosome-related genes, whereas *purple boxes* highlight gene categories involved in amino acid biosynthesis and metabolism. **b**, **c** Specific examples of KEGG pathways whose components are regulated **b** translationally (2-oxocarboxylic acid metabolism) or **c** transcriptionally (tryptophan metabolism). *Red dots* represent genes from the indicated KEGG pathways and the contour plots refer to all genes

To understand the molecular mechanisms behind the increased lifespan of RPKO strains, we compared gene expression of the wild-type strain with that of two RPKO strains with increased (*Δrpl7a* and *Δrpl9a*) and three with decreased (*Δrpl6a*, *Δrpl15b*, and *Δrps27b*) lifespan^18^. For each strain we determined transcript levels by mRNA-seq, ribosome occupancy by ribosome footprint sequencing (Ribo-seq), and protein levels by shotgun proteomics (Supplementary Data 1). Interestingly, all but one RPKO strain (Δ*rpl15b*) showed an increased expression of the paralog of the deleted RP (Supplementary Fig. 1a, b), suggesting that yeast cells can compensate for the lack of individual RP genes. Beyond the expected upregulation of amino acid biosynthesis genes in the large subunit RPKO (RPLKO) strains^26, 27^, we found that RPLKO strains with increased replicative lifespan showed the strongest upregulation of these pathways, both at mRNA and protein levels (Fig. 1a; Supplementary Data 2 for the Kyoto Encyclopedia of Genes and Genomes (KEGG) pathways that are significantly altered in these strains). Unexpectedly, genes from the biosynthetic pathways of phenylalanine, tyrosine, tryptophan, lysine, and arginine and for 2-oxocarboxylic acid metabolism were also upregulated at the level of translation efficiency (Fig. 1a, b), whereas in the alanine, aspartate, glutamate, histidine, and tryptophan metabolism pathways, protein-level changes could be explained by their mRNA-level changes (Fig. 1a, c). Strikingly, the mRNA abundance of genes encoding ribosomal components was strongly reduced in long-lived RPKO strains. This was not compensated by increased translation efficiency on these mRNAs because the protein levels were also reduced (Fig. 1a). Components of the small and large ribosomal subunits were equally affected (Supplementary Fig. 1c), indicating that the repression was not specific to the subunit to which the deleted gene belongs. These data show that deletion of individual RP genes leads to complex changes in gene expression, specifically impacting the abundance and translation efficiency of mRNAs.

### Impaired ribosome assembly in long-lived RPKO strains

**Fig. 2:**
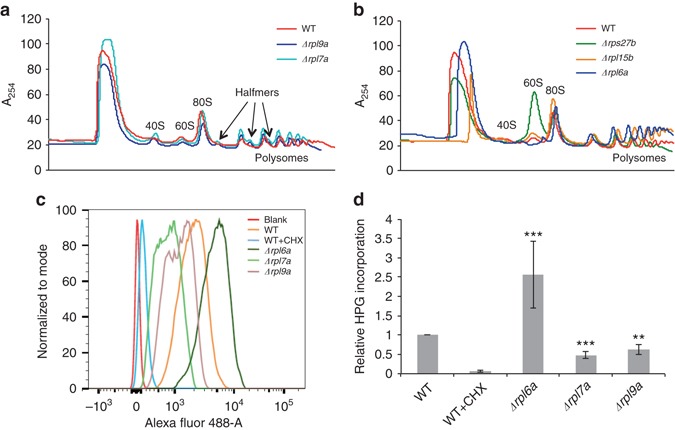
Long-lived RPKO strains show defective ribosome assembly and reduced translation. **a** and **b** Polysome profiles of **a** long-lived and **b** short-lived RPKO strains in relation to the wild-type strain show that the former have a lower 60S-to-40S ratio, and are characterized by the presence of half-mers. **c** Flow cytometry readout for nascent protein synthesis by Click-iT HPG in the different strains and conditions. **d** Quantification of global translation in RPKOs with respect to the wild-type strain. Error bars represent s.d. across three different biological replicates. **p < 0.01, ***p < 0.001. *P*-values were calculated using two-tailed Student’s *t*-test

To evaluate the global impact of RP repression on translation, we generated polysome profiles for all studied strains. The 60S ribosomal subunit was less abundant than the 40S subunit in the long-lived Δ*rpl7a* and Δ*rpl9a* in comparison to the wild-type strain (Fig. 2a; Supplementary Fig. 2a–c), whereas the short-lived strains did not show this pattern (Fig. 2b; Supplementary Fig. 2a, d–f). The long-lived Δ*rpl7a* and Δ*rpl9a* strains yielded half-mer peaks after each monosome/polysome (Fig. 2a; Supplementary Fig. 2b, c), diagnostic for the presence of 48S initiation complexes on actively translated mRNAs^30^. These results indicate that long-lived RPKO strains exhibit delayed/impaired ribosome assembly.

An expected consequence of ribosome assembly defects is a reduced translational output. To directly quantify global translation, we measured the incorporation of the methionine analog l-homopropargylglycine (HPG) in newly synthesized proteins with a fluorimetric assay. These data confirmed that translation was significantly reduced in the Δ*rpl7a* and Δ*rpl9a* strains and increased in Δ*rpl6a* in comparison to wild type (Fig. 2c, d), thereby providing evidence for an association between translation repression and longevity in RPKO strains.

### Generalized uORFs skipping in long-lived RPKO strains

**Fig. 3:**
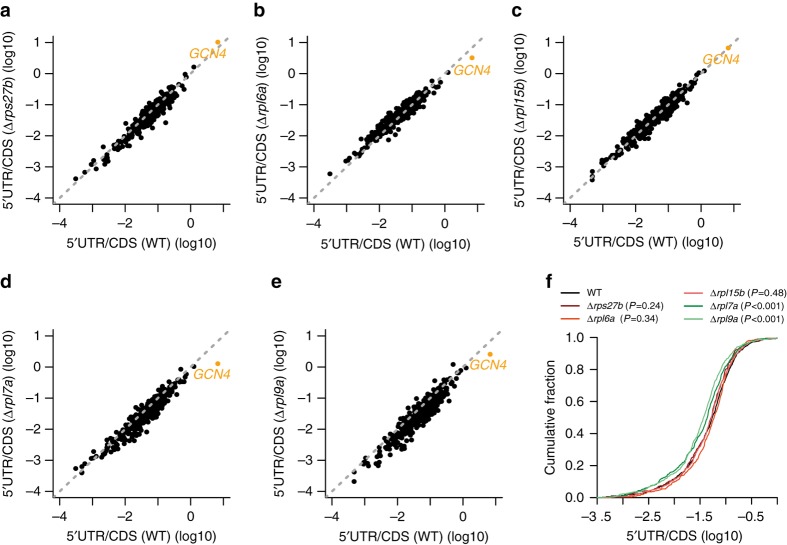
Generalized uORFs skipping in long-lived RPKO strains. **a**–**e** 5′UTR-to-CDS Ribo-seq reads ratio for **a** Δ*rps27b*, **b** Δ*rpl6a*, **c** Δ*rpl15b*, **d** Δ*rpl7a*, and **e** Δ*rpl9a* strains compared to the wild-type strain. Each dot corresponds to a gene containing at least one uORF. The *GCN4* gene is highlighted in *orange*. **f** Cumulative distribution functions for the different strains studied indicate that only long-lived strains show a significant decrease in the 5′UTR-to-CDS ratio compared to the wild-type strain. The comparison of the distributions of ratios between the different RPKO and the wild type was performed with the Mann–Whitney *U* test and the *P*-values for the two-tailed test are indicated. See Supplementary Fig. 3 for the similar analysis of uORF-to-CDS ratios

To determine whether the defective ribosome assembly in long-lived strains is accompanied by a global increase in uORF skipping, we computed the relative ribosome occupancy of 5′ UTRs and corresponding ORFs (or coding sequences, CDS) for the 2067 uORF-containing *S. cerevisiae* genes^31^. Only the long-lived strains with defective ribosome assembly had a lower 5′UTR-to-CDS ratio than the wild type, indicating less occupancy at uORFs (Fig. 3). We obtained similar results when considering only Ribo-seq reads mapping to uORFs instead of to the entire 5′ UTR (Supplementary Fig. 3). Although one may expect that generalized uORF skipping in long-lived RPKO strains leads to an increased translation efficiency of the downstream ORFs, we found little change in the translation efficiency of most genes containing uORFs. Furthermore, the very few genes with a significant change in translation efficiency were either up- or down-regulated (Supplementary Fig. 4). Our data thus indicate that uORF skipping is a general feature of long-lived RPKO strains, and that uORFs skipping rarely has a strong influence on the translation efficiency of the corresponding CDS.

### *GCN4* translation is upregulated in long-lived RPKO strains

**Fig. 4:**
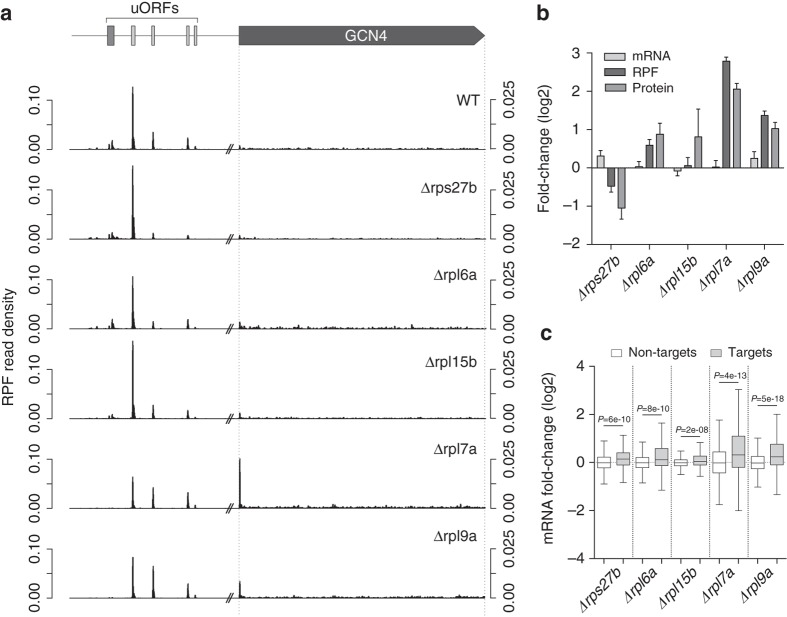
*GCN4* is translationally upregulated in long-lived RPKO strains. **a** Density of ribosome protected fragment (RPF) reads along the *GCN4* locus in the different strains studied. Long-lived strains show decreased density specifically at non-canonical (*dark gray*) and subsequent, first canonical uORF (*light gray*) as well as increased density in the main ORF, particularly at the start. Note the different scales upstream and within the coding regions. **b** Quantification of mRNA, ribosome-protected fragments and protein fold-changes for Gcn4 in the different RPKO strains with respect to the wild-type strain. Error bars indicate the s.e.m. **c** Boxplots show the mRNA fold-changes for Gcn4 targets (*gray*, from http://www.yeastgenome.org) and non-targets (*white*) in the RPKO strains compared to wild-type strain. *P*-values were calculated using the two-sided Mann–Whitney *U* test. Boxes extend from the 25th to 75th percentiles (interquartile range (IQR)), horizontal lines represent the median, whiskers indicate the lowest and highest datum within 1.5*IQR from the lower and upper quartiles, respectively

The *GCN4* transcript showed by far the largest change in the 5′UTR-to-CDS ratio in long-lived strains compared to wild type (Fig. 3d, e). The ribosome occupancy of the *GCN4* locus in the wild-type strain revealed all four well-described inhibitory uORFs in the *GCN4* 5′ UTR^29^, as well as the non-canonical uORF observed more recently^32^ (Fig. 4a). However, it was only in the long-lived Δ*rpl7a* and Δ*rpl9a* strains that the non-canonical uORF and uORF1 were less covered by reads, while the ribosome density strongly increased at the start of the *GCN4* CDS. The mRNA-seq data showed that the increased ribosome occupancy of the CDS is not due to higher mRNA abundance, and targeted proteomics confirmed that it leads to increased protein levels (Fig. 4b). Gcn4 was also increased in the short-lived Δ*rpl6a* strain, albeit less than in the long-lived strains (Fig. 4b). Consistent with an increased Gcn4 level, the mRNA-seq data showed that the expression of its transcriptional targets is also most significantly increased in the long-lived Δ*rpl7a* and Δ*rpl9a* strains compared to wild type, whereas expression of non-targets is not changed (Fig. 4c). These analyses confirm that Gcn4 is translationally upregulated and its known targets are transcriptionally upregulated in long-lived RPKO strains.

### Gcn4 overexpression increases replicative lifespan

**Fig. 5:**
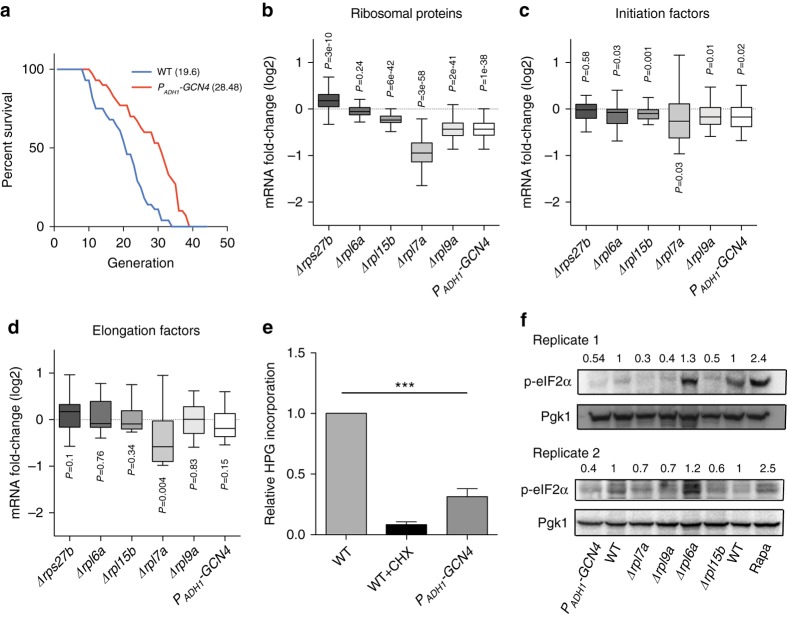
Gcn4 overexpression increases replicative lifespan and dampens global translation independent of eIF2α phosphorylation. **a** The replicative lifespan assay shows that the *GCN4* overexpression strain exhibits a ~45% increase in the mean number of generations compared to the wild-type strain. Mean lifespan values are shown in parentheses. **b**–**d** Boxplots illustrating the distribution of mRNA fold-change of **b** ribosomal proteins, **c** translation initiation factors, and **d** translation elongation factors in RPKOs and *GCN4* overexpression strains relative to the wild-type strain. *P*-values were calculated using the two-sided Mann–Whitney *U* test to compare mRNA fold-changes of genes belonging to a given category (RPs, Initiation factors or Elongation factors) and that of all other genes. Boxes extend from the 25th to 75th percentiles, horizontal lines represent the median, whiskers indicate the lowest and highest datum within 1.5*IQR from the lower and upper quartiles, respectively. **e** Quantification of global translation by Click-iT HPG shows that *GCN4* overexpression strain has a significantly reduced global protein synthesis. Error bars represent s.d. across three different biological replicates. ***p < 0.001. *P*-values were calculated using two-tailed Student’s *t*-test. **f** Quantification of eIF2α phosphorylation through western blot. Two different replicates are shown along with their quantification. Rapamycin vs. vehicle-treated WT was used as positive control for antibody

Although deletion of *GCN4* in RPKO strains partially restores lifespan to wild type levels^18^, whether the Gcn4 overexpression is sufficient to increase yeast replicative lifespan is not known. To test this, we replaced the endogenous promoter and 5′UTR sequence of the *GCN4* gene with the constitutively active *ADH1* promoter. The resulting *P*_*ADH1*_*-GCN4* strain had significantly longer lifespan in comparison to the wild type (mean number of divisions = 28.48 vs. 19.6, based on 30 and 28 cells, respectively, *p* < 0.001, Wilcoxon Rank-sum test; Fig. 5a). This 45% increase in lifespan is comparable to those observed in RPKO strains, which were ~26% for Δ*rpl9a* and ~39% for Δ*rpl7a*^18^. Thus, the overexpression of *GCN4* is sufficient to promote longevity in yeast.

### Gcn4 dampens global translation

To elucidate the molecular mechanism underlying the extended lifespan conferred by Gcn4 overexpression, we measured the global gene expression changes in the *P*_*ADH1*_*-GCN4* strain by mRNA-seq. As expected, multiple pathways involved in amino acid biosynthesis were significantly upregulated in this strain relative to wild type (Supplementary Fig. 5a). Surprisingly, the abundance of genes encoding RPs (Fig. 5b), translation initiation factors (IFs, Fig. 5c) and elongation factors (EFs, Fig. 5d) was reduced (Supplementary Data 3) to the level observed in the long-lived RPKO strains. The general repression of genes encoding components of the translation machinery could lead to a global decrease in translation. Quantifying nascent protein synthesis with the HPG fluorometric assay, we found that global translation rate was indeed decreased in the *P*_*ADH1*_*-GCN4* strain in comparison to wild type (Fig. 5e). The translational repression was not specific to the manner of Gcn4 overexpression, as it was also evident in a strain overexpressing *GCN4* from a galactose-inducible plasmid (*P*_*Gal1-10*_*-GST-GCN4*), and in another strain, carrying a genomically integrated, copper-inducible promoter upstream of the *GCN4* gene (*P*_*Cup1*_*-GCN4*; Supplementary Fig. 5b, c).

To test whether Gcn4 overexpression triggers a stress response, which in turn would lead to general translational repression, we measured the level of phosphorylated eIF2α, a well established stress marker^33^. We did not find an increase, but rather a decrease in the level of eIF2α phosphorylation in the long-lived RPKO and *P*_*ADH1*_*-GCN4* strains (Fig. 5f; Supplementary Fig. 6). These data demonstrate that the global translational repression in Δ*rpl7a*, Δ*rpl9a*, and *P*_*ADH1*_*-GCN4* does not depend on the canonical eIF2α pathway.

### Distinct regulatory motifs in Gcn4-activated/repressed genes

**Fig. 6:**
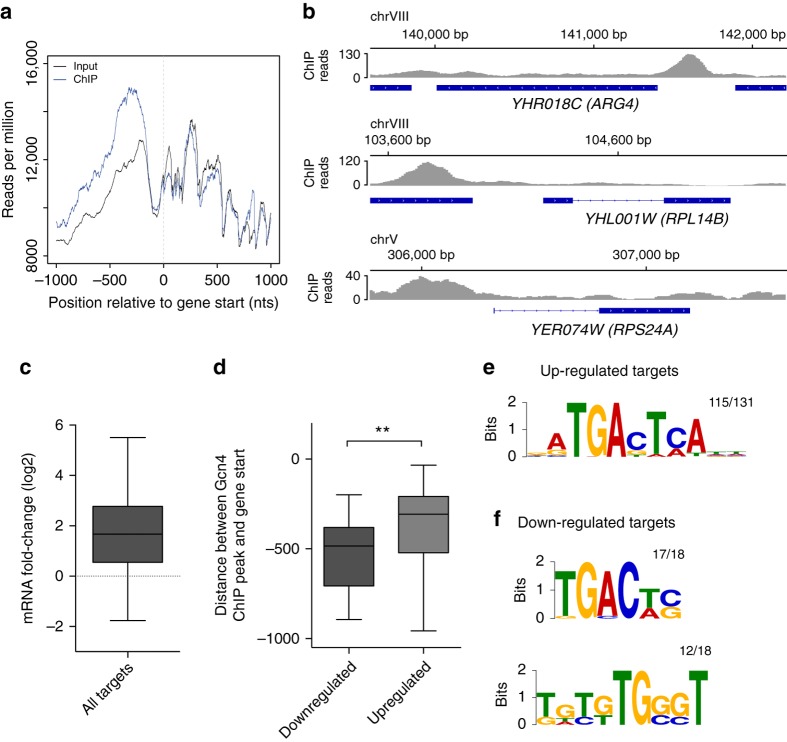
Gcn4-ChIP targets that are activated/repressed upon Gcn4 overexpression have distinct configurations of regulatory elements. **a** Profile of ChIP-seq reads in the 2 kb region centered on the start of the ORF shows an enrichment of Gcn4-ChIP signal over the input signal in the upstream region of the ORF. **b** Example profiles of ChIP-seq reads for three different genes: one related to amino acid biosynthesis (*ARG4*), and two ribosomal proteins (*RPL14B* and *RPS24A*). **c** Boxplot illustrating mRNA fold-change for Gcn4-ChIP targets in the *P*_*Gal1-10*_*-GST-GCN4* strain relative to the respective wild-type strain. The box extends from the 25th to 75th percentiles, the horizontal line represents the median, whiskers indicate the lowest and highest datum within 1.5*IQR from the lower and upper quartiles, respectively. **d** Distances between Gcn4-binding sites and gene starts are significantly higher for repressed compared to activated targets. Boxes extend from the 25th to 75th percentiles, the *horizontal line* represents the median, whiskers indicate the lowest and highest datum within 1.5*IQR from the lower and upper quartiles, respectively. **p < 0.01. *P*-value was calculated using the two-tailed Mann–Whitney *U* test. **e** and **f** Sequence logo for the ChIP peaks associated with **e** upregulated and **f** downregulated genes in the *P*_*Gal1-10*_*-GST-GCN4* strain. The number of peaks where the motif was found out of all the peaks considered is indicated

To determine whether Gcn4 controls directly the transcription of genes encoding components of the translation machinery, we performed chromatin immunoprecipitation followed by high-throughput sequencing (ChIP-seq) in the *P*_*Gal1-10*_*-GST-GCN4* strain, in which *GCN4* was tagged with glutathione S-transferase (GST). We found a strong enrichment of reads in the genomic regions upstream of start codons in the Gcn4-ChIP sample compared to the input chromatin (Fig. 6a; Supplementary Fig. 7a). We identified 327 ChIP peaks, of which 151 could be unambiguously assigned to downstream genes (Supplementary Data 4). Although only 25.8% of these ChIP-inferred targets were previously reported as Gcn4-responsive genes (Supplementary Fig. 7b), all of the ChIP targets, including the 74.2% that were not identified before, contained high-scoring Gcn4-binding sequences (Supplementary Fig. 7c).

To evaluate the transcriptional response of Gcn4-ChIP targets, we analyzed gene expression in the *P*_*Gal1-10*_*-GST-GCN4* strain, that was used for the ChIP analysis, and in the corresponding wt-*URA3* control. We observed consistent changes in gene expression upon *GCN4* overexpression in *P*_*Gal1-10*_*-GST-GCN4* and *P*_*ADH1*_*-GCN4* cells (Pearson’s correlation *R* = 0.65, *p* < 0.001). Out of the 149 unambiguous Gcn4-ChIP targets whose expression could be detected, 131 were upregulated and 18 were downregulated, suggesting that Gcn4 acts as a transcriptional activator as well as repressor (Fig. 6c). The upregulated targets were mainly amino acid biosynthesis genes (Supplementary Fig. 7d) and had the Gcn4-ChIP peaks at ~250 nucleotides upstream of the translation start (Fig. 6d). The downregulated genes did not share a specific molecular pathway and had the Gcn4-ChIP peaks farther upstream. Whereas RP genes were generally repressed upon Gcn4 overexpression (Fig. 5b), only a few contained Gcn4-ChIP peaks in their promoters (Fig. 6b), indicating that most RP genes are indirectly repressed by Gcn4. Specifically, two of the directly repressed, unambiguous Gcn4-ChIP targets were RPs. In addition, in contrast to most of the Gcn4 targets, translation-related factors had reduced expression, regardless of the method used for Gcn4 overexpression (Supplementary Fig. 7e).

To confirm that Gcn4 interacts directly with the promoters of the downregulated targets, we searched for overrepresented sequence motifs in the Gcn4-ChIP peaks with the MEME software^34^. Surprisingly, although 88% of the upregulated Gcn4 targets exhibited the canonical Gcn4-binding motifs^35–37^, all but one of the downregulated targets had a shorter form of the motif (Fig. 6e, f). In addition, the majority of the downregulated targets (12 out of 18, ~67%) contained Rap1 binding motifs^38^ (Fig. 6f), and half of these (6 out of the 12 promoter regions) have been previously reported to be regulated by Rap1^25, 39–42^. The frequency of the Rap1 motif at upregulated targets of Gcn4 was much lower (~17%). Also unexpectedly, the Gcn4-binding sites are located downstream of the predicted Rap1 binding sites in upregulated Gcn4 targets, whereas in downregulated targets the configuration is inverted (Supplementary Fig. 8).

Mutation of the Gcn4 DNA-binding domain was reported to impair the upregulation of genes in amino acid biosynthetic pathway but not the repression of RP genes^43^. However, we found that a strain that overexpressed the genomically integrated S242L Gcn4 mutant showed both reduced upregulation of amino acid biosynthetic pathways as well as de-repressed RP gene expression (Supplementary Fig. 9). Collectively, our data demonstrate that more than 10% of genes with Gcn4-ChIP peaks are repressed and that the DNA-binding domain of Gcn4 is necessary for both its activating and repressing effects on gene expression.

### Gcn4 generally represses translation

**Fig. 7:**
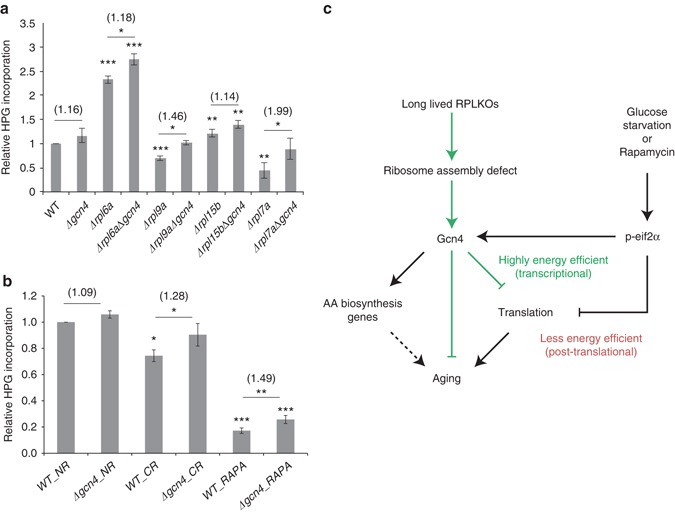
Gcn4 strongly represses translation in the long-lived RPKO strains and stresses. Quantification of global translation by Click-iT HPG for **a** different single and double KO strains, **b** glucose-starved (CR) and rapamycin-treated (RAPA) yeast cells. *GCN4* deletion restores translation to the level of the wild-type strain in RPKO strains and also leads to increased translation in stressed cells. The significance of the two-tailed *t*-test between any given deletion strain and the wild-type strain is depicted above the respective bar. Mean values of the relative translation change between the *GCN4* deletion strain and the respective parental strain are shown in parentheses. Error bars represent s.d. across three different biological replicates except glucose starvation where *n* = 2. The *p*-value for the two-tailed t-test is indicated by ‘*’: *p < 0.05, **p < 0.01, ***p < 0.001. **c** Model for Gcn4 effect on translation and aging. *Green lines* indicate the findings in this study, *black continuous lines* denote previously established links, and the *dashed line* indicates a connection that remains to be studied

The above findings strongly suggest that the induction of Gcn4 contributes to the decreased protein synthesis capacity in long-lived RPKO strains. To test this, we deleted *GCN4* and measured the translation rate in the wild type and single RPKO strains. We found that deletion of *GCN4* leads to increased translation in all strains, the largest changes occurring in the long-lived RPKO strains (Fig. 7a). These results indicate that Gcn4 is required for the reduced translational output of long-lived RPKO strains. To determine whether Gcn4 generally represses translation beyond the RPKO strains, we measured the rate of protein synthesis in wild-type strains treated with rapamycin or subjected to glucose starvation. These conditions have been shown to induce Gcn4 expression and reduce translation^44, 45^. The protein synthesis assay showed that indeed, rapamycin treatment and glucose starvation reduce translation (Fig. 7b). Importantly, deletion of *GCN4* significantly mitigated this effect. Altogether, these results indicate that translation repression is a general function of Gcn4.

## Discussion

Recent studies have demonstrated that yeast strains with individual RP gene deletions differ widely in replicative lifespan, and that longevity is partially dependent on Gcn4 expression^11, 18^. However, the mechanism by which Gcn4, a transcriptional activator of amino acid biosynthesis genes, influences lifespan, is unknown. Here we uncovered a general function of Gcn4 in repressing translation. This finding has important implications for the coupling between stress, protein synthesis, and longevity.

For our study we chose the wild-type strain, as well as strains with increased (Δ*rpl7a* and Δ*rpl9a*) or decreased lifespan (Δ*rpl6a*, Δ*rpl15b*, and Δ*rps27b*)^18^. Although amino acid biosynthesis genes were upregulated in all RPLKO strains^26, 27^, the upregulation was most pronounced in the long-lived strains, in line with the strongest induction of Gcn4 in these strains. Long-lived RPLKO strains also showed downregulation of translation-related genes (Fig. 1a), and a consistently impaired protein synthesis (Fig. 2). Polysome profiling revealed half-mers (Fig. 2a) diagnostic for the presence of the 48S initiation complex on actively translated mRNAs as a result of delayed monosome formation^30, 46^. As fast assembly of 80S ribosomes at *GCN4* uORFs prevents Gcn4 protein production, the slow assembly of 80S explains the pronounced Gcn4 upregulation in long-lived RPKO strains^26, 27^ (Figs. 3 and 4). Consistent with this interpretation, profiling of genome-wide ribosome occupancy revealed generalized uORF skipping in the strains displaying half-mers (Fig. 3). Incidentally, although one might expect that increased uORF skipping generally leads to increased translation of the downstream ORF, our data does not support this hypothesis. The translation efficiency of most genes with uORFs remained unchanged and for the few genes that showed significant changes, we observed both up- and down-regulation (Supplementary Fig. 4). This is in line with observations that uORFs are not always inhibitory^31^.

Strikingly, the overexpression of Gcn4 was sufficient to repress protein synthesis and also to downregulate the expression of translation-related genes (Fig. 5). Furthermore, Gcn4 overexpression did not lead to increased eIF2α phosphorylation (Fig. 5f, Supplementary Fig. 6), showing that repression of translation-related genes that follows Gcn4 overexpression can be decoupled from the program triggered by cellular stress. The Gcn4-dependent repression of RPs expression has been described before, under conditions of amino acid starvation^22^. As most RP genes do not have a Gcn4-binding motif in their promoters, it was concluded that this response, although Gcn4-dependent, is indirect, perhaps through squelching of transcription factors that are required for RP gene expression^22, 47^. More recently, it was proposed that the RP downregulation upon amino acid starvation is the result of Gcn4 displacing Esa1 from the RP-activating Esa1-Rap1 complex^43^. Here we found that the DNA binding activity of Gcn4 is necessary for RP gene repression (Supplementary Fig. 9), which remains consistent with the squelching hypothesis. Nevertheless, we also found that Rap1 and Gcn4-binding motifs co-occur in the promoters of genes that are repressed upon Gcn4 overexpression (Fig. 6f), indicating that both of these proteins bind to the promoters of repressed genes in a sequence-specific manner. Although the frequency of Rap1 binding motifs in the promoters of upregulated Gcn4 targets is much lower, there are some upregulated Gcn4 targets that also co-targeted by Rap1. What accounts for the differences in expression changes among genes whose promoters contain both Gcn4 and Rap1 binding motifs remains to be further analyzed. Here we observed that the Rap1 binding sites are preferentially located upstream of the Gcn4-binding sites in the upregulated targets, whereas they are located downstream in the downregulated targets (Supplementary Fig. 8). In addition, the Gcn4 motif that we inferred from downregulated targets is shorter than the motif we inferred from the upregulated targets.

Since reduced translation is linked to increased lifespan^13, 15, 48^, our findings provide a compelling explanation for the effect of Gcn4 on lifespan. Consistently, the knockout of *GCN4* almost entirely restored global translation to wild type levels in the long-lived RPKO strains (Fig. 7a). Notably, the magnitude of Gcn4 induction (Fig. 4b), the corresponding repression of RP genes (Fig. 5b), and the reduction in the translation capacity (Fig. 2d), all correlated with the increase in lifespan. However, Gcn4 also increases amino acid biosynthesis, and so far, these two activities could not be decoupled. Uncovering the precise mechanism by which Gcn4 represses the expression of translation factors is likely necessary to be able to assess the relative contribution of these two activities in increasing the replicative lifespan.

In physiological contexts such as environmental stress^49^, expression of Gcn4 follows eIF2α phosphorylation, which also leads to reduced protein synthesis^33^. Why would Gcn4 and phosphorylated eIF2α act simultaneously to globally repress translation? As ribosome biogenesis consumes a large fraction of a cell’s energy^20^, transcriptional regulation of translation-related genes by Gcn4 is an energy efficient mechanism to globally inhibit protein synthesis (Fig. 7c), which may operate synergistically with the repression of the translation process through the phosphorylation of eIF2α.

An interesting open question is how certain mRNAs encoding amino acid biosynthesis genes are translationally upregulated in long-lived RPKO strains despite globally impaired protein synthesis capacity (Fig. 1b). It may be that the corresponding transcripts are selectively regulated by mRNA-binding factors^50^. Alternatively, the deletion of the RP gene could confer a specialized function to cellular ribosomes lacking this protein, namely increased affinity to specific mRNAs and hence enhanced translation^51^.

Gcn4 is a highly conserved protein and its mammalian homolog is known as activating transcription factor-4 (ATF4). Similarly to *GCN4*, translation of *ATF4* upon stress or amino acid starvation is regulated through uORFs^52, 53^. Also similarly to Gcn4, Atf4 binds directly to the promoters of translation-related genes in mouse embryonic fibroblasts^54^. Unlike Gcn4, however, Atf4 has context-dependent effects on cellular and organism lifespan. Upon ER stress, ATF4 induction leads to increased protein synthesis and cell death^54^. In contrast, in cellular models of Parkinson’s disease, ATF4 protects against neuronal cell death^55^ and its increased expression has also been observed in slow-aging mice^56, 57^. Our study provides a compelling molecular basis for the effect of Gcn4 and perhaps its mammalian counterpart ATF4 on longevity, relying on the transcriptional repression of protein synthesis genes.

## Methods

### Yeast strains and growth

The yeast strains used in this study are listed in the Supplementary Table 1. All yeast strains are in the *BY4741* genetic background. All single RPKOs and wild-type *BY4741* strains were obtained from GE Dharmacon as a part of the haploid yeast ORF deletion collection. The *P*_*GAL1/10*_*-GST-GCN4* strain was also purchased from GE Dharmacon. Other strains were generated by standard genetic techniques^58^. The chromosomal promoter exchange for *P*_*ADH1*_*-GCN4* and *P*_*CUP1*_*-GCN4* strains were performed according to refs. ^59, 60^. Yeast was grown on YPD (1% yeast extract, 2% peptone, and 2% glucose) medium at 30 °C, 200 r.p.m. unless otherwise stated. The cells were collected in mid-log phase at OD_600_ 0.4–0.8.

### mRNA sequencing

Libraries for mRNA sequencing were prepared for three biological replicates. A yeast cell pellet was resuspended in 1 ml of lysis buffer from Dynabeads mRNA DIRECT Kit (61011, Life technologies) and lysed at 4 °C with 1 volume of acid washed glass beads in a FastPrep instrument (Thermo Scientific) using 2 cycles with the following settings: 45 s at 6.5 speed with 3 min pause on ice between cycles. Further, poly(A) + RNA was isolated directly from cell lysate using the Dynabeads mRNA DIRECT Kit according to manufacturer’s protocol. Libraries for mRNA sequencing were prepared using the “directional mRNA-seq sample preparation” protocol from Illumina, with minor modifications. In brief, after isolation, 50 ng of mRNA was chemically fragmented by incubating the mRNA solution with twice the volume of alkaline hydrolysis buffer (50 mM sodium carbonate [NaHCO_3_/Na_2_CO_3_] pH 9.2, 1 mM EDTA) at 95 °C for 5 min to obtain fragments of ~200–300 bases. The fragmented mRNA was immediately purified with RNeasy MinElute Cleanup Kit (74204, Qiagen) to stop the reaction and to remove small RNA fragments (<100 bases). Further, purified, fragmented mRNA was treated with thermosensitive alkaline phosphatase FastAP (EF0651, Fermentas) at 37 °C for 30 min and then at 75 °C for 5 min to inactivate FastAP. The fragmented mRNA was further incubated with ATP and T4 polynucleotide kinase (EK0032, Fermentas) at 37 °C for 1 h and subsequently purified. Ligation of RNA 3′ adapter (RA3, part # 15013207, Illumina) was done using T4 RNA Ligase 2, truncated K227Q (M0351L, New England Biolabs Inc) according to the Illumina protocol. The ligation step was followed by RNA purification as described above to remove unligated 3′ adapters. The RNA 5′ adapter (RA5, part #15013205, Illumina) was ligated using T4 RNA ligase (EL0021, Fermentas) according to the Illumina protocol, and the RNA was then purified to remove unligated 5′ adapters. Complementary DNA (cDNA) was synthesized using RNA RT Primer (RTP, part #15013981, Illumina) and SuperScript III (18080044, Invitrogen) as per Illumina protocol. Libraries were amplified for 14 cycles of PCR using forward (RNA PCR Primer (RP1), part #15005505 Illumina), and reverse (Illumina PCR Primer, Index) PCR primers. Reverse PCR primers with different indexes were used to prepare libraries from different samples thereby enabling multiplexed sequencing. Libraries were sequenced for 51 cycles on an Illumina HiSeq 2000 instrument.

### Polysome profiling and Ribo-seq

Polysome profiling and sequencing of ribosome-protected mRNA fragments were performed for three biological replicates (except for Δ*rpl6a*, for which only two replicates were obtained) according to protocol described in ref. ^61^. In brief, yeast cells were treated for 1 min with 100 μg per ml of cycloheximide (CHX) to stabilize the translating ribosomes on mRNA. Cells were harvested by vacuum filtration, flash frozen in liquid nitrogen, and later lysed under cryogenic conditions in lysis buffer (20 mM Tris HCl, pH 7.4, 150 mM NaCl, 5 mM MgCl_2_, 1 mM DTT, 1% Triton X-100, and 100 μg/ml cycloheximide) using freezer mill (Spex). Lysate was centrifuged at 3000×*g* for 3 min at 4 °C and then at 10,000×*g* for 5 min at 4 °C, to clarify the lysate. A fraction of lysate equivalent to *A*_260_ = 10 was treated with 6 μl of RNase I (100 U per μl, Ambion) for 45 min at room temperature (RT) with gentle agitation for ribosome profiling and RNaseI was inactivated by addition of 10 µl of SuperaseIn (20U per µl). Notably, for polysome profiling, the lysate was not treated with RNaseI but taken directly for subsequent steps. 7–47% linear sucrose gradient was prepared in 50 mM Tris-HCl (pH = 7.5), 50 mM NH4Cl, 12 mM MgCl2, 0.5 mM DTT, and 100 µg per ml CHX using Gradient Master instrument (Biocomp) according to the manufacturer’s instruction. The samples were loaded on the precooled linear gradient and centrifuged at 35,000 rpm for 3 h at 4 °C in a TH-641 rotor (Thermo Scientific). For ribosome profiling only different fractions of the gradient were collected in 1% SDS solution using a Density Gradient Fractionation System (Brandel) with a setting of pump speed (0.75 ml per min) and collection time 32 sec per tube, then flash frozen.

The appropriate fractions that contain monosomes were processed for footprint library preparation according to ref. ^62^. In brief, RNA was isolated from the collected monosomes fraction with the phenol chloroform method. RNA fragments of appropriate size (28–30 nt) were selected by running samples on 15% polyacrylamide denaturing TBE-Urea gel and visualized by SYBR Gold (Life Technologies). Size-selected RNA was dephosphorylated by T4 polynucleotide kinase (PNK, New England Biolabs) treatment for 1 h at 37 °C. PNK was heat inactivated and RNA was purified using phenol chloroform method and overnight precipitation of RNA in ethanol. Preadenylated 3′ linker was ligated to dephosphorylated RNA by using T4 RNA ligase 2, truncated (New England Biolabs). The ligation reaction was carried out for 4 h at 22 °C. The ligation reaction was run on 15% polyacrylamide denaturing TBE-Urea gel to separate and purify the ligated from the unligated product and from unused 3′ linker. Gel purified, ligated RNA was reverse transcribed by Superscript III (Invitrogen) for 30 min at 48 °C in a total reaction volume of 20 µl. After reverse transcription, the RNA was hydrolyzed by adding 2.2 µl of 1 N NaOH solution and incubating for 20 min at 98 °C. First-strand cDNA was further gel purified by electrophoresis on 15% polyacrylamide denaturing TBE-Urea gel and circularized by incubating with CircLigase II ssDNA Ligase (Epicentre) for 60 min at 60 °C, followed by inactivation of CircLigase by heating at 80 °C for 10 min. Thereafter, circular cDNA was PCR-amplified and then the amplified products were gel purified on 8% native polyacrylamide gel. The prepared library was sequenced on an Illumina platform.

### Quantitative proteomics

Quantitative proteomics was performed for three biological replicates according to the protocol described below in a step by step manner.

### Sample preparation

For each sample, 108 yeast cells were resuspended in 100 µl lysis buffer (2% sodium deoxycholate, 100 mM ammonium bicarbonate), sonicated for 2 × 10 s using a vial tweeter and spinned down. A small aliquot of the supernatant was taken to determine the protein concentration using a BCA assay (Thermo Fisher Scientific). Aliquots containing 50 µg proteins were taken from each sample, respectively, reduced with 5 mM TCEP for 15 min at 95 °C and alkylated with 10 mM iodoacetamide for 30 min in the dark at 25 °C. After quenching the reaction with 12 mM N-acetyl-cysteine the samples were diluted with 100 mM ammonium bicarbonate buffer to a final DOC concentration of 1%. Proteins were digested by incubation with sequencing-grade modified trypsin (1/50, w/w; Promega, Madison, WI, USA) overnight at 37 °C. Then, the samples were acidified with 2 M HCl to a final concentration of 50 mM, incubated for 15 min at 37 °C and the precipitated detergent removed by centrifugation at 10,000×*g* for 15 min. Subsequently, peptides were desalted on C18 reversed-phase spin columns according to the manufacturer’s instructions (Microspin, Harvard Apparatus) and dried under vacuum. The dried peptide samples were subsequently labeled with isobaric tag (TMT 6-plex, Thermo Fisher Scientific) according to the manufacturer’s instructions. The pooled sample was again desalted on C18 reversed-phase spin columns according to the manufacturer’s instructions (Macrospin, Harvard Apparatus) and dried under vacuum. In total, three pooled TMT samples containing one biological replicates of all six conditions, respectively, were generated.

### Off-Gel electrophoresis

The TMT labeled samples was resolubilized to a final concentration of 1 mg/ml in Off-Gel electrophoresis buffer containing 6.25% glycerol and 1.25% IPG buffer (GE Healthcare). The peptides were separated on a 12 cm pH 3–10 IPG strip (GE Healthcare) with a 3100 OFFGEL fractionator (Agilent) as previously described^63^ using a protocol of 1 h rehydration at maximum 500 V, 50 μA and 200 mW. Peptides were separated at maximum 8000 V, 100 μA and 300 mW until 20 kVh were reached. Subsequently, neighboring fractions were pooled (1&2, 3&4 …11&12) and the thus generated 6 peptide fractions were desalted using C18 reversed-phase columns according to the manufacturer’s instructions (Microspin, Harvard Apparatus), dried under vacuum and subjected to LC-MS/MS analysis.

### Mass spectrometric analysis

The setup of the μRPLC-MS system was as described previously^64^. Chromatographic separation of peptides was carried out using an EASY nano-LC 1000 system (Thermo Fisher Scientific), equipped with a heated RP-HPLC column (75 μm × 50 cm) packed in-house with 1.9 μm C18 resin (Reprosil-AQ Pur, Dr. Maisch). Aliquots of 1 μg total peptides were analyzed per LC-MS/MS run using a linear gradient ranging from 95% solvent A (0.15% formic acid, 2% acetonitrile) and 5% solvent B (98% acetonitrile, 2% water, 0.15% formic acid) to 30% solvent B over 180 min at a flow rate of 200 nl/min. Mass spectrometry analysis was performed on a dual pressure LTQ-Elite Orbitrap mass spectrometer equipped with a nanoelectrospray ion source (both Thermo Fisher Scientific). Each MS1 scan was followed by high-collision-dissociation (HCD, both acquired in the Orbitrap) of the 10 most abundant precursor ions with dynamic exclusion for 60 s. Total cycle time was ~2 s. For MS1, 10^6^ ions were accumulated in the Orbitrap cell over a maximum time of 300 ms and scanned at a resolution of 60,000 FWHM (at 400 *m*/*z*). MS2 scans were acquired at a target setting of 50,000 ions, accumulation time of 100 ms and a resolution of 15,000 FWHM (at 400 *m*/*z*). Singly charged ions and ions with unassigned charge state were excluded from triggering MS2 events. The normalized collision energy was set to 35%, and one microscan was acquired for each spectrum.

### Database searching and protein quantification

The acquired raw-files were converted to the mascot generic file (mgf) format using the msconvert tool (part of ProteoWizard, version 3.0.4624 (2013-6-3)). Using the MASCOT algorithm (Matrix Science, Version 2.4.0), the mgf files were searched against a decoy database containing normal and reverse sequences of the predicted SwissProt entries of Saccharomyces cerevisiae (www.uniprot.org, release date 20/10/2014) and commonly observed contaminants (in total 13,386 protein sequences) generated using the SequenceReverser tool from the MaxQuant software (Version 1.0.13.13). The precursor ion tolerance was set to 10 ppm and fragment ion tolerance was set to 0.01 Da. The search criteria were set as follows: full tryptic specificity was required (cleavage after lysine or arginine residues unless followed by proline), 2 missed cleavages were allowed, carbamidomethylation (C), TMT6plex (K and peptide N-terminus) were set as fixed modifications and oxidation (M) as a variable modification. Next, the database search results were imported to the Scaffold Q+software (version 4.3.3, Proteome Software Inc., Portland, OR, USA) and the protein false identification rate was set to 1% based on the number of decoy hits. Specifically, peptide identifications were accepted if they could be established at >93.0% probability to achieve an FDR <1.0% by the scaffold local FDR algorithm. Protein identifications were accepted if they could be established at >5.0% probability to achieve an FDR <1.0% and contained at least 1 identified peptide. Protein probabilities were assigned by the Protein Prophet program^65^. Proteins that contained similar peptides and could not be differentiated based on MS/MS analysis alone were grouped to satisfy the principles of parsimony. Proteins sharing significant peptide evidence were grouped into clusters. Acquired reporter ion intensities in the experiments were employed for automated quantification and statically analysis using a modified version of our in-house developed SafeQuant R script^66^. In brief, reporter ion intensities were corrected for isotopic impurities according to the manufacturer’s instructions. Intensities for each peptide and protein identification were summed, globally normalized across all acquisition runs and employed for ratio calculation and statistical analysis. Additionally, ratio distortion was controlled using spiked in protein calibrants as recently described^67^. The correlation between biological replicates was 0.872–0.911 (median 0.886), indicating that our estimates of protein abundance have a good reproducibility.

### Translation assay

To quantify nascent protein synthesis as a measure of global translation, we followed the protocol of non-radioactive metabolic labelling assay kit “Click-iT HPG Alexa Fluor 488 Protein Synthesis Assay Kit” (Thermo Fisher Scientific). The method is based on the incorporation of L-HPG, an amino acid analog of methionine containing an alkyne moiety, and Alexa Fluor 488 azide. The signal intensity of incorporated HPG-Alexa Fluor 488 was measured by flow cytometry. Mean fluorescence intensities were computed from 10,000–50,000 cells of each strain and then normalized by the mean fluorescence intensity of the wild-type strain. We integrated the *MET15* gene in all the strains used for the translation assay to allow the growth of cells in medium lacking methionine, as BY4741 strains are auxotrophic for this amino acid.

### Replicative lifespan assay

Yeast replicative lifespan assays were performed as described previously^68^. In brief, yeast was grown on YPD agar plates at 30 °C and virgin daughter cells were isolated. Thereafter, virgin daughters were allowed to grow and divide while daughter cells were microdissected using a conventional manual microdissector (MSM, Singer Instruments) until mother cells stopped dividing. The differences in mean replicative lifespan among strains were compared with the Wilcoxon Rank-sum test.

### Western blot

Yeast cells were lysed in 300 µl RIPA buffer containing protease inhibitor and phosphatase inhibitor as described in mRNA sequencing protocol above. 15–25 µg total protein was resolved on 10% SDS PAGE. For probing expression of p-eIF2α and Pgk1 with the respective antibodies used at 1:1000 dilution (Cell Signaling #3597 and Thermo Fisher Scientific #459250), we followed the protocol from Cell Signaling for transfer, blocking, incubation, washing, and developing the membrane. As positive control, we included samples from the wild-type strain treated for 30 min with rapamycin (200 ng per ml) and with the equivalent volume of solvent (ethanol) that we used to dissolve the rapamycin.

### ChIP-seq

The ChIP protocol was adapted from ref. ^69^. 500 ml of yeast cells grown to mid-log phase were crosslinked in fixing buffer (50 mM HEPES pH 7.5, 1 mM EDTA pH 8.0, 0.5 mM EGTA pH 8.0, 100 mM NaCl, and 1% formaldehyde) for 10 min with continuous rocking at RT, and then quenched with 125 mM glycine for 5 min. Cells were washed three times with cold PBS and collected. Nuclei were isolated and lysed to obtain crosslinked chromatin. Simultaneously, the antibody was coupled with protein G magnetic beads (10004D, Thermo Fisher Scientific) by incubating 100 μl of protein G beads with 10 μg of anti-GST antibody (27-4577-01, GE Healthcare Life Sciences) for minimum 1 h at RT with continuous rotation. A probe sonicator was then used in cold conditions to reduce heating, for 6 cycles of 30 s pulse-on at amplitude value of 60 and 1 min, and 15 s pulse-off to obtain chromatin fragments of 100–500 bp, followed by centrifugation at 20,000×*g* for 10 min at 4 °C to remove nuclear debris. Further, 3% chromatin from each sample was kept as input control and an equal amount (~0.75–1 mg) of chromatin was incubated with magnetic beads-coupled antibody at 4 °C overnight, with continuous rotation. Immuno-complexes were washed with 1 ml of wash buffers as described in the original protocol. Samples of washed immuno-complexes along with the input were further treated with RNase and then with proteinase K followed by overnight reverse crosslinking at 65 °C with continuous shaking at 1400 rpm, in a thermoblock with heating lid. DNA was purified using Ampure (Beckman Coulter) beads as detailed in ref. ^69^.

Libraries of ChIPed and input DNA were prepared according to the instruction manual of NEBNext ChIP-Seq Library Prep Reagent Set from Illumina. In brief, end repair of input and ChIPed DNA was done by incubating with T4 DNA Polymerase Klenow fragment and T4 PNK enzyme at 20 °C for 30 min. The reaction was purified using Ampure beads according to the instruction manual. An A nucleotide overhang at the 3′ end was produced by treating the end-repaired DNA with dATP and Klenow Fragment (3′ → 5′ exo^–^) at 37 °C for 20 min followed by DNA purification. Double-stranded DNA adapters were ligated to the dA overhang DNA by T4 DNA ligase reaction at 37 °C for 30 min, the DNA was purification and size-selected as described in the instructions manual. Size-selected DNA was PCR-amplified for 16 cycles using NEBNext High-Fidelity 2X PCR Master Mix with Illumina universal forward primer and indexed reverse primer, that enabled multiplexing of samples for sequencing. Amplified DNA was finally purified and sequenced on an Illumina Hiseq2500 instrument.

### uORF analyses

Yeast mRNAs with annotated uORFs with ATG, GTG, or TTG initiation codons were retrieved from ref. ^31^. Ribo-seq 5′ UTR and CDS library-normalized counts were aggregated across the multiple replicates for the same strain to maximize the number of genes amenable for the follow-up analyses. Then, for all the genes containing more than 50 reads in both the 5′UTR and CDS for any given RPKO and wild-type strain, the ratio of 5′ UTR-to-CDS counts was calculated. We have also estimated the uORF-to-CDS ratio, counting only reads mapped within a predicted uORF (i.e., an open reading frame within the 5′ UTR that starts with ATG, GTG, or TTG as initiation codon), rather than in the entire 5′ UTR.

### mRNA- and Ribo-seq analysis

mRNA-seq reads were first subjected to 3′ adapter trimming (5′-TGGAATTCTCGGGTGCCAAGG-3′) and quality control (reads shorter than 20 nucleotides or of low quality, i.e., for which over 10% of the nucleotides had a PHRED quality score <20, were discarded) using the FASTX-toolkit (http://hannonlab.cshl.edu/fastx_toolkit/). Segemehl^70^ (version 0.1.7–411) was used to map reads to the yeast transcriptome, allowing a minimum mapping accuracy of 90%. CDS annotations were taken from the yeast database (http://www.yeastgenome.org) and 5′/3′UTR annotations from ref. ^71^.

For Ribo-seq, the procedure was similar to the one used above with only two alterations: (1) the sequence of the 3′ adapter that was used and trimmed was 5′-CTGTAGGCACCATCAAT-3′; (2) only reads mapped to coding regions were counted toward differential expression analysis. The Ribo-seq reads had the expected length (28–32nt) and for each read length, the relative location of the P site with respect to the read start was inferred as the value for which the correct position of the start codon and the 3-nt periodicity was most apparent (the number of reads at the first frame being larger than at both other frames). Only read lengths showing the expected 3-nt periodicity along the CDS were considered for further analyses. Note that beyond the enrichment at translation start, no strong bias along the open reading frame was observed (Supplementary Fig. 10).

For both types of data, transcript counts were calculated based on uniquely mapped reads, for all considered read lengths, and used for differential expression with DESeq2^72^. The fold-change in translation efficiency was calculated by dividing the Ribo-seq fold-change by the mRNA-seq fold-change. Up/down-regulation was considered significant when the mRNA or RPF abundance changed more than 2-fold between strains and when the corresponding False Discovery Rate was lower than 0.01.

Three biological replicates were obtained for each strain and each sample type (with the exception of Δ*rpl6a*, which only has two biological replicates for the Ribo-seq data) and were used for the estimation of differential expression. Pearson correlations between replicates of 0.966–0.999 (median of 0.981) for RNA-seq and 0.992–0.9997 (median of 0.998) for Ribo-seq indicate that our data have very good reproducibility.

### ChIP-seq analysis

ChIP-seq reads were first subject 3′ adapter trimming (5′-TGGAATTCTCGGGTGCCAAGG-3′) and quality control using the FASTX-toolkit (http://hannonlab.cshl.edu/fastx_toolkit/). Reads were then mapped to the yeast genome (sacCer3) using Segemehl^70^ (version 0.1.7–411), allowing a minimum mapping accuracy of 90%. ChIP peaks were found with MACS^73^ (version 1.4.2) using the mapped reads from input and Gcn4-ChIP samples as follows:   macs14 -t gcn4_chip.sam -c input.sam -f SAM -g 2e7 -w -n gcn4.chip.output

We used bedtools to find peaks whose summits overlapped with 1 kb regions upstream of the annotated CDS starts of yeast and thereby annotated the Gcn4 targets. Peaks that could be unambiguously associated with only one gene were selected for further analyses, and the respective genes were reported as Gcn4 targets. Finally, we used MEME^74^ to find enriched sequence motifs embedded in the ChIP peaks ([−50,50] nucleotides around peak summits) associated with genes that were found either up or downregulated upon Gcn4 overexpression using the following command:   meme chip_peaks.fa -dna -maxsize 60000 -mod zoops -nmotifs 3 -minw 6 -maxw 20 -revcomp

### Motif scoring in promoter regions

We used Patser (version 3b; http://genetics.wustl.edu/gslab/resources/) parameterized with the position-dependent frequency matrix of Gcn4^75^ and Rap1^38^ to predicted binding sites for these two transcription factors in promoter regions (i.e., regions located 1 kb upstream of start codons). As the score of a given promoter we took the highest score of a predicted binding site in the entire promoter region.

### Gene-set analyses

For each gene set present in KEGG (http://www.kegg.jp) containing at least 10 genes, we computed the mean fold-change across all the genes between a given RPKO strain and the wild-type strain. The list of RP genes was retrieved from the KEGG database (sce03010), whereas the initiation and elongation factors were retrieved from the GO repository (GO:0006413 and GO:0006414, respectively) in the yeast database (http://www.yeastgenome.org). Gcn4 literature targets were retrieved from the yeast database (http://www.yeastgenome.org). KEGG enrichment analyses for up and downregulated genes were performed with GeneCodis (http://genecodis.cnb.csic.es) using the default parameters and all yeast genes as the reference list.

### Data availability

All sequencing data have been deposited to Gene Expression Omnibus (GEO) under accession number GSE85591. The MS proteomics data have been deposited to ProteomeXchange with the identifier PXD004760.

## Electronic supplementary material

Supplementary Information

Supplementary Data 1

Supplementary Data 2

Supplementary Data 3

Supplementary Data 4
