# Supplementary material for: The Gcn4 transcription factor reduces protein synthesis capacity and extends yeast lifespan
Source: Nat Commun. 2017 Sep 6;8:457. doi: 10.1038/s41467-017-00539-y (PMC5587724; doi:10.1038/s41467-017-00539-y)
Supplement: Supplementary file 1 — Supplementary Information [file 41467_2017_539_MOESM1_ESM.pdf]

Description of Supplementary Files

File Name: Supplementary Information
Description: Supplementary Figures, Supplementary Table.

File Name: Supplementary Data 1
Description: mRNA, translation efficiency and protein fold-changes for RPKO strains.

File Name: Supplementary Data 2
Description: Statistical significance of differences in the expression of different KEGG pathway. Values represent -log10(p-value) (values greater than 2 are colored in red) resulting from the Mann Whitney U test comparing the fold-change of the genes belonging to a given KEGG pathway and that of all other genes.

File Name: Supplementary Data 3
Description: mRNA fold-changes for translation machinery genes.

File Name: Supplementary Data 4
Description: Gcn4-ChIP targets.

Supplementary Table 1. Yeast strains used in the study

strain name	genotype	strain description	Reference
wt	*BY4741 MATa his3Δ1 leu2Δ0 met15Δ0 ura3Δ0*		[1]
Δrpl6a	*BY4741 MATa his3Δ1 leu2Δ0 met15Δ0 ura3Δ0 rpl6a::kanMX4*		[1]
Δrpl7a	*BY4741 MATa his3Δ1 leu2Δ0 met15Δ0 ura3Δ0 rpl7a::kanMX4*		[1]
Δrpl9a	*BY4741 MATa his3Δ1 leu2Δ0 met15Δ0 ura3Δ0 rpl9a::kanMX4*		[1]
Δrpl15b	*BY4741 MATa his3Δ1 leu2Δ0 met15Δ0 ura3Δ0 rpl15b::kanMX4*		[1]
Δrpl27b	*BY4741 MATa his3Δ1 leu2Δ0 met15Δ0 ura3Δ0 rps27b::kanMX4*		[1]
wt	*BY4741 MATa his3Δ1 leu2Δ0 ura3Δ0*	*MET15* reintegration	this study
Δrpl6a	*BY4741 MATa his3Δ1 leu2Δ0 ura3Δ0 rpl6a::kanMX4*	*MET15* reintegration	this study
Δrpl7a	*BY4741 MATa his3Δ1 leu2Δ0 ura3Δ0 rpl7a::kanMX4*	*MET15* reintegration	this study
Δrpl9a	*BY4741 MATa his3Δ1 leu2Δ0 ura3Δ0 rpl9a::kanMX4*	*MET15* reintegration	this study
Δrpl15b	*BY4741 MATa his3Δ1 leu2Δ0 ura3Δ0 rpl15b::kanMX4*	*MET15* reintegration	this study
Δrpl27b	*BY4741 MATa his3Δ1 leu2Δ0 ura3Δ0 rps27b::kanMX4*	*MET15* reintegration	this study
P_{ADH1}-*GCN4*	*BY4741 MATa his3Δ1 leu2Δ0 ura3Δ0 gcn4::kanMX4-promADH1-GCN4*	PCR insert from pYM-N6	this study
P_{ADH1}-*GCN4*^{S242L}	*BY4741 MATa his3Δ1 leu2Δ0 ura3Δ0 gcn4::kanMX4-promADH1-GCN4(S242L)*	Mutation insertion by Delitto perfetto method	this study

P_{CUP1}-GCN4	*BY4741 MATa his3Δ1 leu2Δ0 ura3Δ0 gcn4::kanMX4-promCUP1-GCN4*	PCR insert from pYM-N10	this study
$P_{GAL1-10}$-GST-GCN4	*BY4741 MATa his3Δ1 leu2Δ0 met15Δ0 ura3Δ0 pEGH 2μ URA3 promGAL1-10-GST-GCN4*		[2]
wt-*URA3*	*BY4741 MATa his3Δ1 leu2Δ0 met15Δ0 ura3Δ0 pRS426 2μ URA3*		this study
ΔGCN4	*BY4741 MATa his3Δ1 leu2Δ0 ura3Δ0 gcn4::CloneNAT*	*pFA6a-CloneNAT*	this study
Δrpl6aΔGCN4	*BY4741 MATa his3Δ1 leu2Δ0 ura3Δ0 rpl6a::kanMX4 gcn4::CloneNAT*	*pFA6a-CloneNAT*	this study
Δrpl7aΔGCN4	*BY4741 MATa his3Δ1 leu2Δ0 ura3Δ0 rpl7a::kanMX4 gcn4::CloneNAT*	*pFA6a-CloneNAT*	this study
Δrpl9aΔGCN4	*BY4741 MATa his3Δ1 leu2Δ0 ura3Δ0 rpl9a::kanMX4 gcn4::CloneNAT*	*pFA6a-CloneNAT*	this study
Δrpl15bΔGCN4	*BY4741 MATa his3Δ1 leu2Δ0 ura3Δ0 rpl15b::kanMX4 gcn4::CloneNAT*	*pFA6a-CloneNAT*	this study

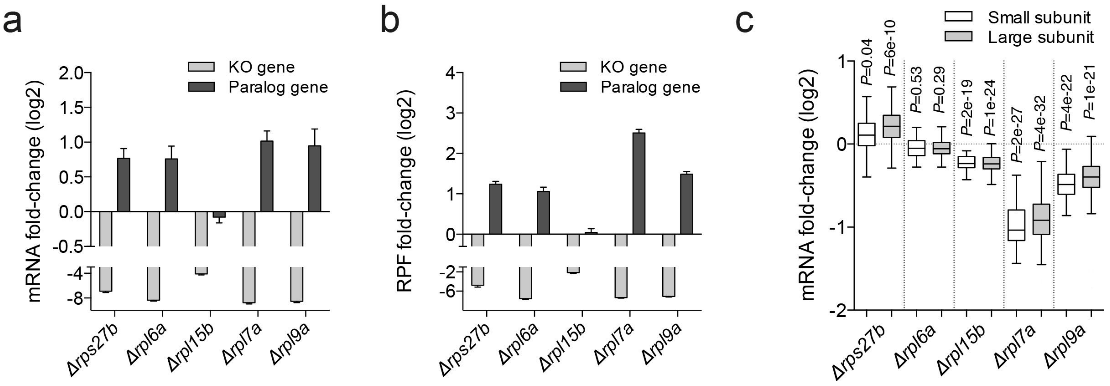

Supplementary Fig. 1. Gene expression regulation in RPKO strains

(a) Fold-changes of mRNA and (b) ribosome protected fragment (RPF) reads derived from RPKO genes (light gray) and their corresponding paralogs (dark gray). Error bars indicate the standard error of the mean. (c) Boxplots with mRNA fold-changes for RP genes belonging to either the small (white) and large (gray) ribosomal subunit. Shown are also the P-values in the two-sided Mann Whitney U test comparing mRNA fold-changes of genes belonging to a given ribosomal subunit (small or large) and those of all other genes. Boxes extend from the 25th to 75th percentiles (interquartile range (IQR)), horizontal lines represent the median, whiskers indicate the lowest and highest datum within 1.5*IQR from the lower and upper quartiles, respectively.

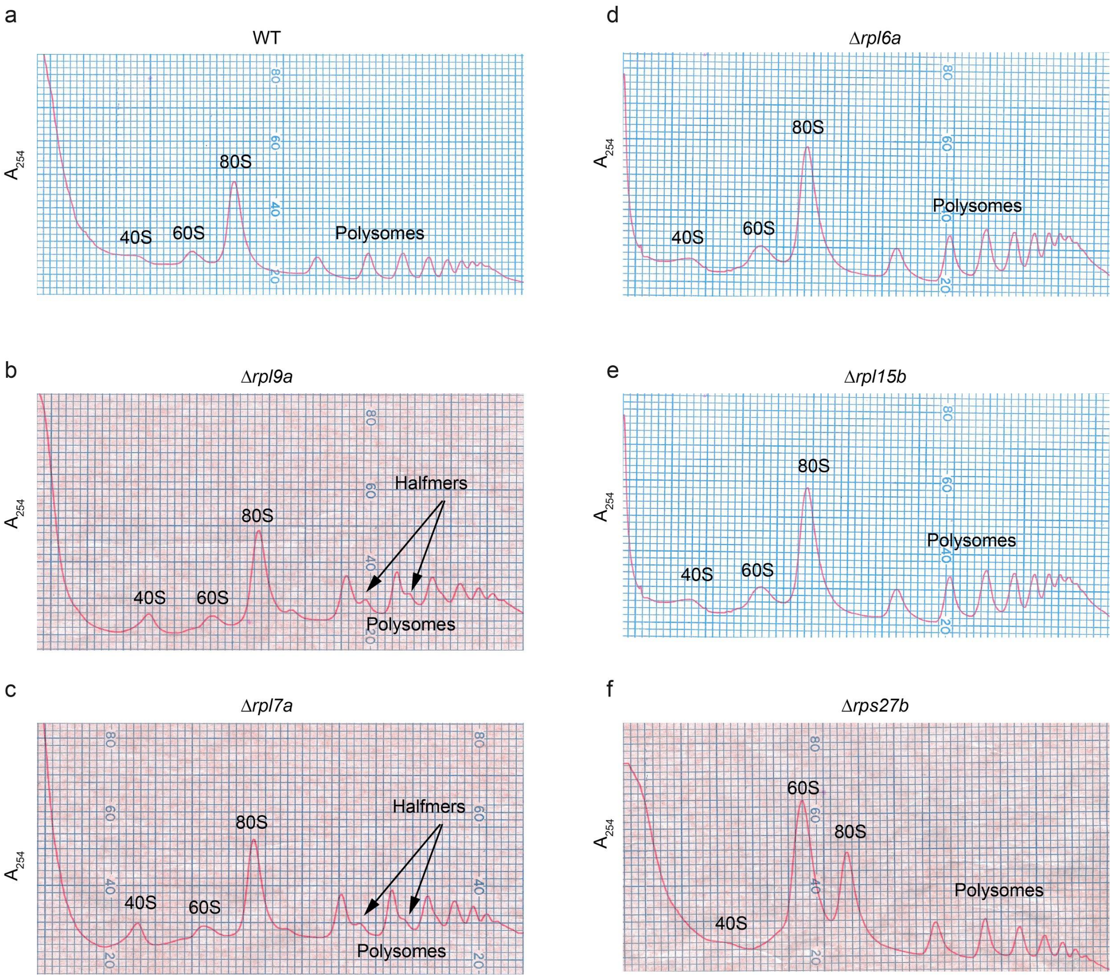

Supplementary Fig. 2. Polysome profiles reveal features of translation in individual strains

(a to f) Representative polysome profiles for (a) WT, (b and c) long-lived RPKOs and (d, e and f) short-lived RPKOs. The profiles of long-lived strains show the presence of half-mers (b and c), lower 60S-to-40S ratio, and reduced number of distinguishable polysomes peaks compared to the wild type strain (a). Short-lived strains resulting from deletion of components of the large ribosomal subunit show higher monosome and polysome peaks (d and e) compared to the wild type strain (a). A notable increase in the 60S-to-40S ratio was registered in the short-lived strain resulting from deletion of an RP from the small subunit (f).

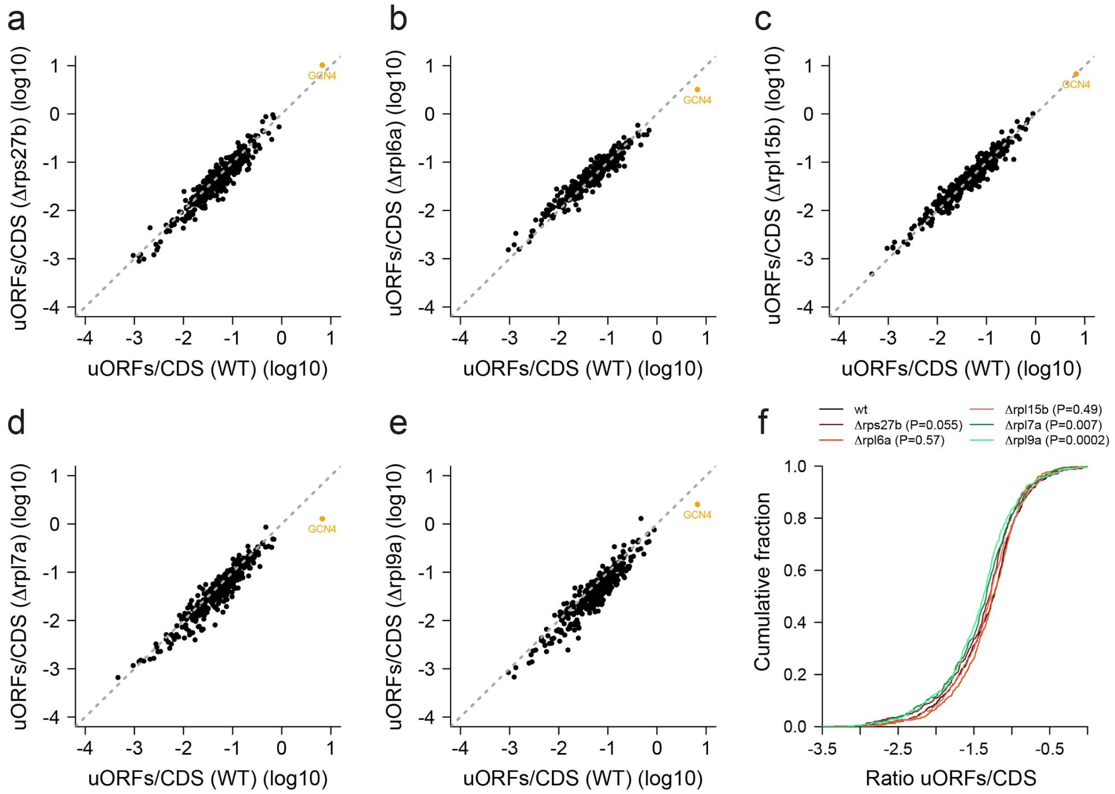

Supplementary Fig. 3. Generalized uORFs skipping in long-lived RPKO strains
(a to e) Ration of uORFs-to-CDS Ribo-seq reads for (a) *Δrps27b*, (b) *Δrpl6a*, (c) *Δrpl15b*, (d) *Δrpl7a* and (e) *Δrpl9a* strains compared to the wild type strain. Each dot corresponds to a gene containing at least one uORF. The *GCN4* gene is highlighted in orange. (f) Cumulative distribution functions for the different strains studied indicate that only long-lived strains show a significant decrease in the uORFs-to-CDS ratio compared to the wild type strain. The Mann Whitney U test was used to compare the distributions of ratios between RPKO and the wild type, and the P-values of the two-tailed test are indicated.

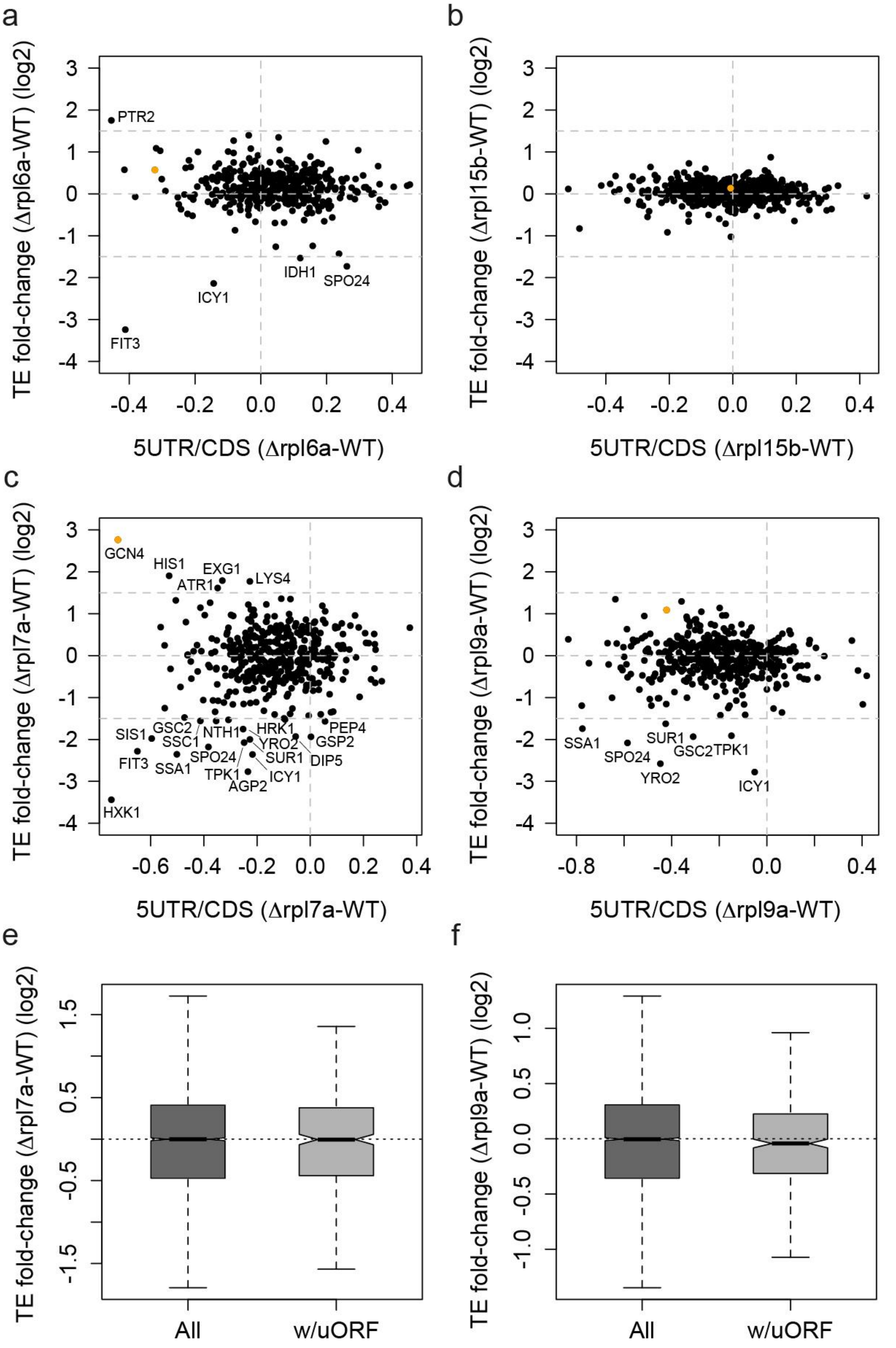

Supplementary Fig. 4. The effect of generalized uORF skipping on translation efficiency

(a to d) Scatter plot of translation efficiency (TE) fold-changes of (a) *Δrpl6a*, (b) *Δrpl15b*, (c) *Δrpl7a* and (d) *Δrpl9a* with respect to the wild type strain, versus the difference in 5'UTR-to-CDS ratios for all genes containing uORFs. *GCN4* is highlighted in orange, and genes showing significant changes ($|\Delta TE| >= 1.5$) in translation efficiency have been labeled. (e and f) Boxplots of TE fold-changes in the long-lived (e) *Δrpl7a* and (f) *Δrpl9a* relative to the wild type strain for all genes (dark gray) and genes containing one or more uORFs (light gray). There are no significant differences in the TE distribution between the two groups. Boxes extend from the 25th to 75th percentiles, horizontal lines represent the median, whiskers indicate the lowest and highest datum within 1.5*IQR from the lower and upper quartiles, respectively. Boxplot notches indicate the 95% confidence interval around the median.

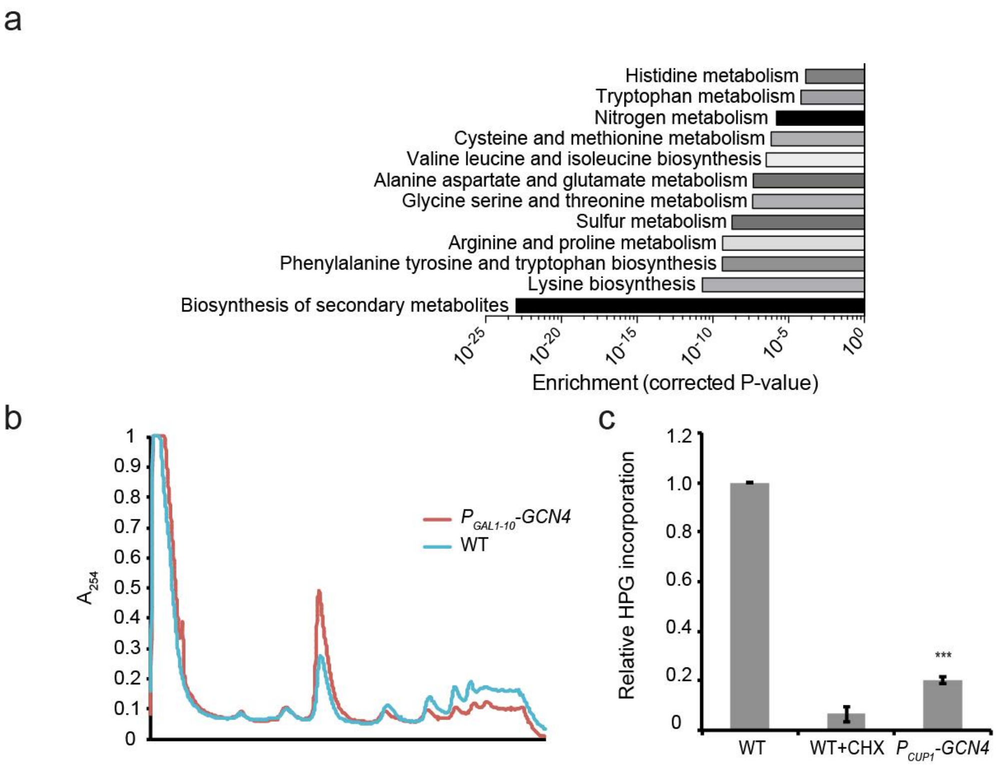

Supplementary Fig. 5. Gene and translation regulation in *GCN4*-overexpressing strains

(a) KEGG pathways that are enriched among genes up-regulated in the *GCN4* overexpressing strain (P_{ADH1}-*GCN4*). (b) Polysome profile for the strain overexpressing *GCN4* from a plasmid ($P_{Gal1-10}$-*GST-GCN4*) and the respective wild type strain. (c) Quantification of global cellular translation in strains with the genomically-integrated copper inducible promoter for the overexpression of *GCN4* (P_{Cup1}-*GCN4*) and the respective wild type strain. Error bars represent standard deviation across three different biological replicates. *** $p < 0.001$. P-values were calculated with the two-tailed student t-test.

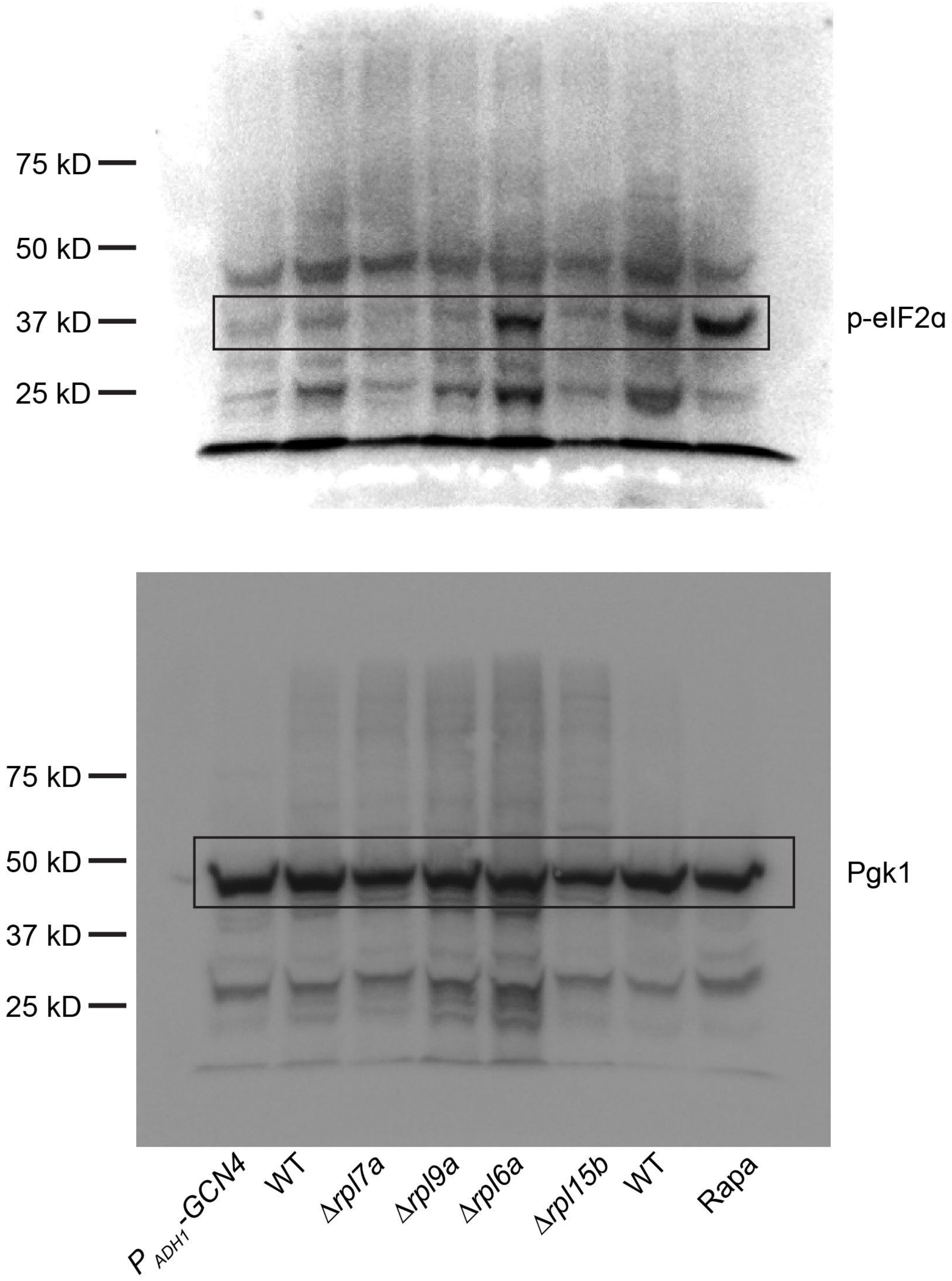

Supplementary Fig. 6. Uncropped images of replicate 1 of western blot result in Fig. 5f

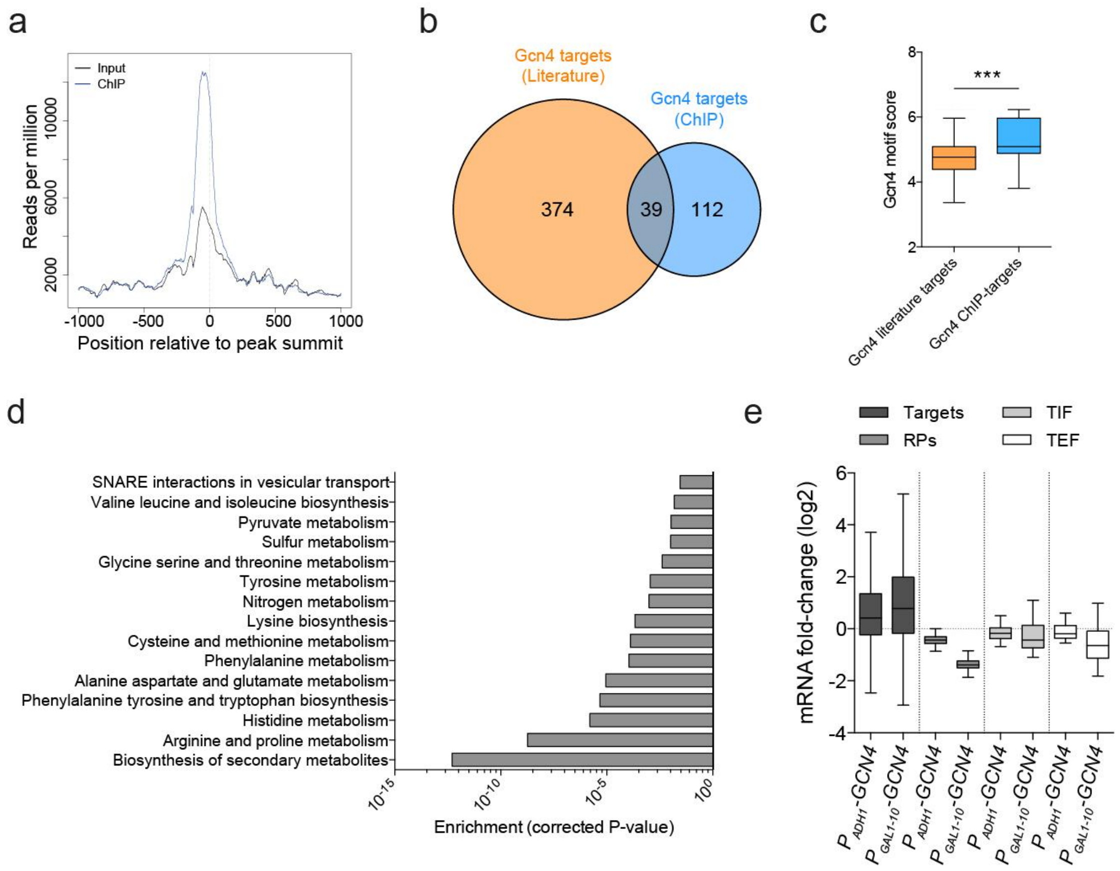

Supplementary Fig. 7. Analysis of Gcn4-ChIP targets

(a) Read coverage +/- 1kb of the ChIP peak summit. There is around a ~3-fold enrichment in reads in the ChIP sample compared to the input sample. (b) Venn diagram depicting the intersection between Gcn4 targets that could unambiguously be associated with a ChIP peak in our study (n = 151) and the Gcn4 targets reported in the literature (n = 413). (c) Gcn4 motif scores of targets inferred by Gcn4-ChIP are significantly higher compared to those of targets from the literature. *** indicates p-value < 0.001 in the two-tailed the Mann Whitney U test. (d) KEGG pathways that were enriched among genes up-regulated in the $P_{Gal1\text{-}10}$-*GST-GCN4*. (e) mRNA fold-changes for all Gcn4 targets from the literature, ribosomal proteins (RPs), translation initiation factors (TIFs), and translation elongation factors (TEFs) in the P_{ADH1}-*GCN4* and $P_{Gal1\text{-}10}$-*GST-GCN4* strains relative to corresponding wild type strains. Boxes extend from the 25th to 75th percentiles, horizontal lines represent the median, whiskers indicate the lowest and highest datum within 1.5*IQR from the lower and upper quartiles, respectively.

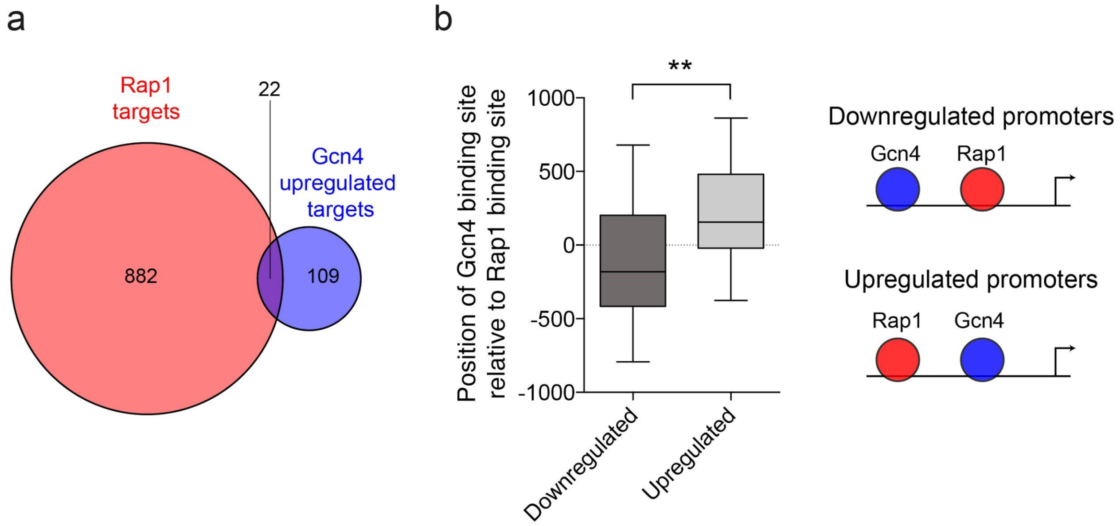

Supplementary Fig. 8. Analysis of putative Gcn4-Rap1 co-regulation

(a) Venn diagram depicting the intersection of previously described Rap1-regulated genes and the upregulated targets of Gcn4 inferred here from ChIP. (b) Position of the Gcn4 binding site relative to Rap1 binding site (in nucleotides, negative values indicate that Gcn4 binding is predicted to be upstream of Rap1, in the direction of transcription) in the upstream regions of downregulated (dark gray) and upregulated (light gray) targets (left). Schematic representation of the architecture of downregulated and upregulated promoters co-targeted by Gcn4 and Rap1 (right). ** indicates p-value < 0.01 in the two-tailed the Mann Whitney U test. Boxes extend from the 25th to 75th percentiles, horizontal lines represent the median, whiskers indicate the lowest and highest datum within 1.5*IQR from the lower and upper quartiles, respectively.

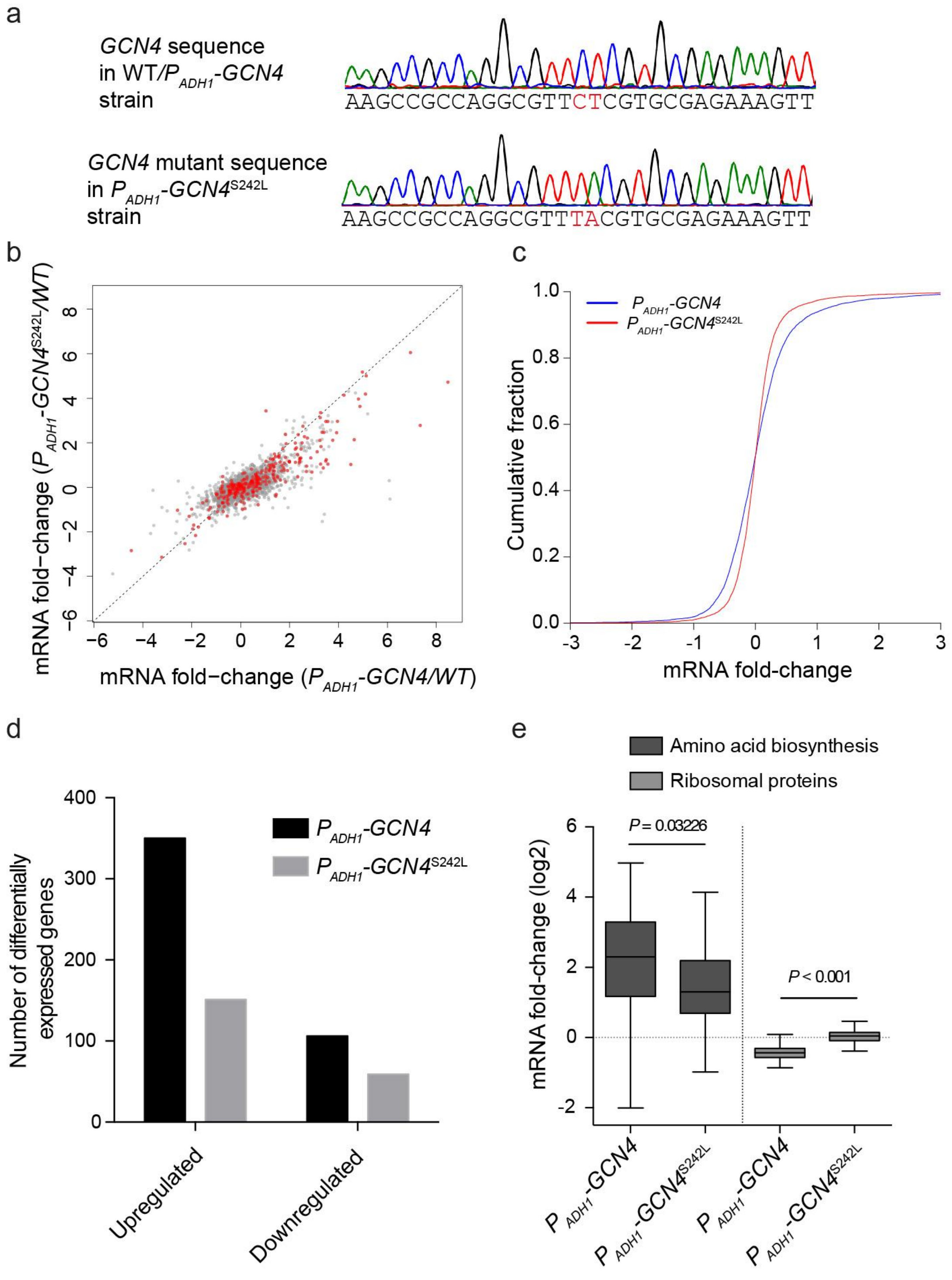

Supplementary Fig. 9. The DNA-binding domain of Gcn4 is required for the repression of RP genes

(a) Chromatogram showing the wildtype *GCN4* genomic sequence and the sequence after mutating two nucleotides to convert Serine to Leucine at position 242 (S242L). The nucleotide that were mutated are shown in red, both in the WT and the mutated sequence. (b) Scatter plot of mRNA fold-changes for the two strains overexpressing either Gcn4 (x-axis) or Gcn4 S242L (y-axis) compared to the wildtype strain. Red dots represent Gcn4 targets from the yeast database. (c) Cumulative distribution functions of mRNA expression changes observed in the two GCN4 overexpression

strains relative to the wild type strain. (d) Number of genes that were differentially expressed in the two strains relative to wildtype (i.e. genes for which the mRNA abundance changed more than 2 fold relative to wildtype at a False Discovery Rate lower than 0.01). (e) Boxplots show the mRNA fold-changes for genes encoding amino acid biosynthesis (dark gray) and ribosomal (light gray) protein genes in the WT and S242L Gcn4-overexpressing strains with respect to wildtype. P-values were calculated using the two-sided Mann Whitney U test. Boxes extend from the 25th to 75th percentiles (interquartile range (IQR)), horizontal lines represent the median, whiskers indicate the lowest and highest datum within 1.5*IQR from the lower and upper quartiles, respectively.

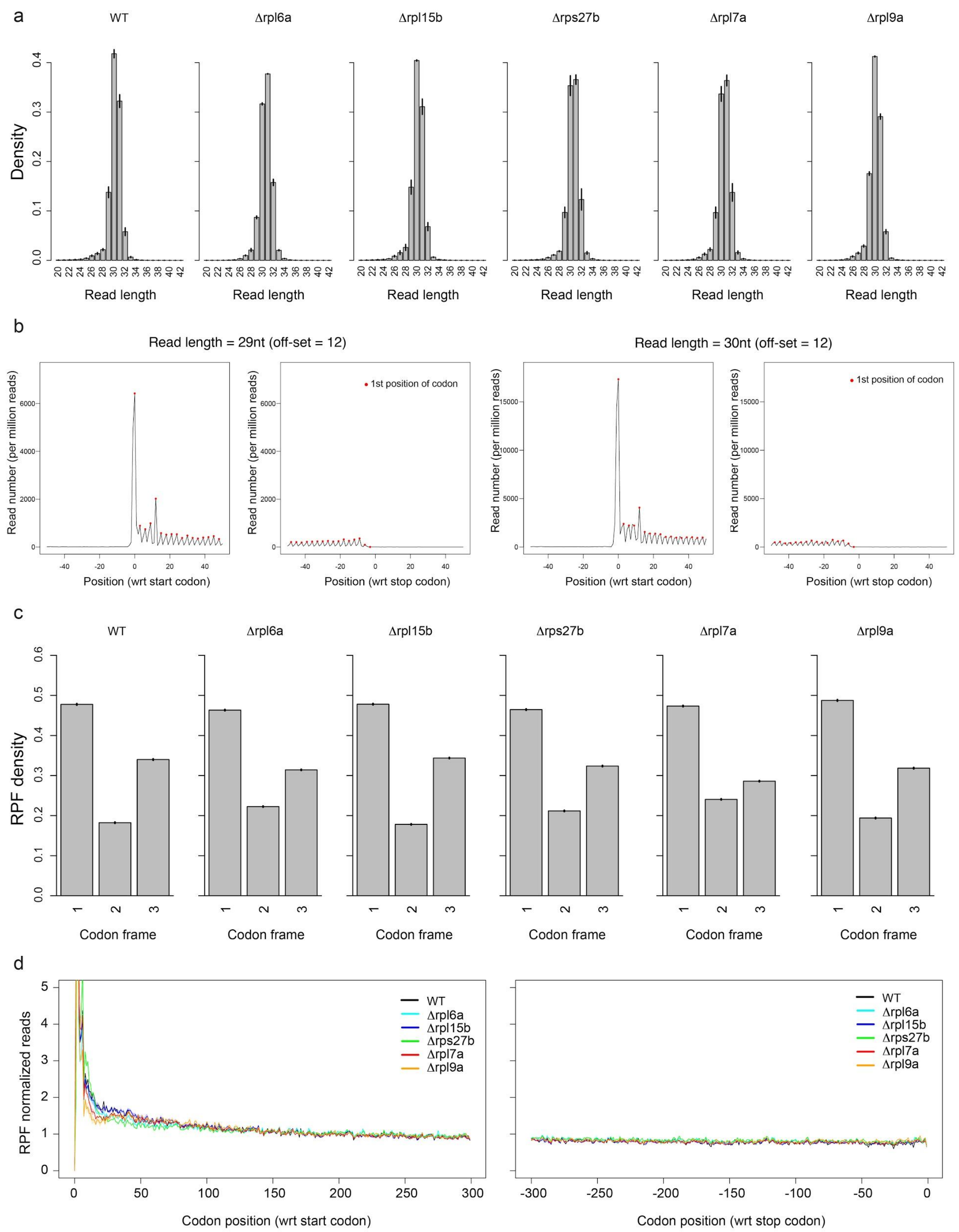

Supplementary Fig. 10. Quality control of ribosome occupancy sequencing data.

(a) RPF read length distribution across the different strains studied. The height of the bar and the error bar show the mean and standard error of the mean, respectively, of read length density across replicates. (b) Metagene analysis of ribosome profiling reads. For each read length, the relative location of the P site with respect to the read start was inferred as the value for which the correct position of the start codon and the 3-nt periodicity was most apparent. The number of reads mapped around start and stop codons is shown for two different read lengths (29nt and 30nt) for the WT strain. The red dots correspond to the first positions of each codon in the CDS. (c) Density of RPF reads starting at each of the three reading frames (1 represents the first position of the

codon). The height of the bar and the error bar show the mean and standard error of the mean, respectively, of the density for all read lengths showing 3-nt periodicity across all the ORFs (depending on the data set, read lengths of 28-34 nucleotides were typically used). (d) Metagene analysis of RPF reads across the different strains studied. RPF reads per codon (A-site) in a given ORF were individually normalized by the mean number of reads within the respective ORF, and then averaged across all the ORFs.
